# Single-cell and spatial transcriptomic profiling reveals distinct immune landscapes in murine lungs infected with H1N1 versus H5N1 influenza viruses

**DOI:** 10.1128/jvi.00746-26

**Published:** 2026-06-29

**Authors:** Qianqian Zhang, Yuying Zhang, Hailiang Sun, Haoning Li, Yao Wang, Fanhua Wei

**Affiliations:** 1 School of Animal Science and Technology, Ningxia University 56693, Yinchuan, China; 2 School of Biological Science and Technology, University of Jinan 12413, Jinan, China; 3 College of Veterinary Medicine, South China Agricultural University, Guangzhou, China; 4 Key Laboratory of Animal Disease Prevention and Control & Veterinary Biosafety of Ningxia, Yinchuan, China; University of Minnesota Twin Cities, Minneapolis, Minnesota, USA

**Keywords:** influenza virus, single-cell RNA sequencing, immune responses, alveolar macrophages, viral pathogenesis

## Abstract

Influenza A viruses of divergent pathogenicity elicit distinct host immune responses, yet the underlying cellular and spatial dynamics remain poorly defined. Here, we integrate single-cell RNA sequencing (scRNA-seq) and spatial transcriptomics to systematically compare the immune landscapes in murine lungs following infection with seasonal H1N1 or a highly pathogenic avian influenza H5N1 virus belonging to clade 2.3.2.1. Analysis of 84,162 immune cells identifies 12 distinct cell types, suggesting that H1N1 infection is associated with robust interferon and inflammatory responses, characterized by alveolar macrophage depletion and extensive infiltration of monocytes and neutrophils. In contrast, H5N1 clade 2.3.2.1 infection is characterized by attenuated interferon signaling, delayed monocyte activation, dysregulated neutrophil maturation, and impaired intercellular communication—features that may be distinct from previously characterized H5N1 strains. Spatial mapping indicates distinct distribution and interaction patterns of immune cells between the two infections. Functional validation through myeloid cell depletion supports the critical role of the monocyte-macrophage axis in disease progression. These findings provide a high-resolution atlas of pulmonary immune responses to influenza viruses of differing pathogenicity and highlight cell-type-specific mechanisms of immune modulation.

**IMPORTANCE:**

Seasonal H1N1 influenza virus causes annual epidemics, while highly pathogenic avian H5N1 virus has a high mortality rate and pandemic potential. Understanding why H5N1 causes more severe disease is critical for developing better treatments. In this study, we used advanced single-cell and spatial technologies to create a detailed map of the immune response in the lungs of mice infected with either H1N1 or a clade 2.3.2.1 H5N1 virus. We discovered that H1N1 triggers a strong, well-organized immune response that controls the virus. In contrast, H5N1 infection leads to a disorganized and weakened response where key immune cells fail to activate and communicate properly. These findings suggest that the H5N1 virus may evade and suppress the host’s immune system. Our study provides a high-resolution immune atlas and identifies potential targets for new therapies against severe influenza.

## INTRODUCTION

Since the early 20th century, four influenza pandemics (1918, 1957, 1968, and 2009) have been caused by influenza A viruses (IAVs). Characterized by high transmissibility, genetic variability, and broad host tropism, IAVs pose a persistent global health threat, infecting nearly one billion people annually and resulting in hundreds of thousands of deaths (http://www.who.int/news-room/fact-sheets/detail/influenza). Among these, zoonotic viruses like the highly pathogenic avian influenza virus (HPAIV) H5N1 are of particular concern due to their increasing spillover into mammalian populations. Among the circulating H5N1 lineages, clade 2.3.2.1 has emerged as one of the predominant variants in avian populations and has been increasingly associated with mammalian spillover events (1). Viruses belonging to this clade exhibit distinct biological features, including enhanced binding to α2,3-linked sialic acid receptors in the lower respiratory tract and the ability to replicate systemically in mammalian hosts (1). Importantly, clade 2.3.2.1 viruses have been responsible for multiple human infections and continue to pose a significant pandemic threat. Despite their public health importance, the immune responses elicited by clade 2.3.2.1 viruses in the mammalian lung remain poorly characterized.

Pulmonary infection by IAV is a leading cause of mortality related to acute lung injury worldwide. Viral entry begins in the upper respiratory tract and progresses to the lungs. Alveolar macrophages (AMs), the predominant resident immune cells in the lower airways, recognize viral pathogen-associated molecular patterns (PAMPs) via pattern recognition receptors (PRRs). Early studies have demonstrated that the highly pathogenic H5N1 virus can infect human AMs, albeit with relatively low efficiency and without excessive pro-inflammatory cytokine induction (2). These cells phagocytose IAV-infected targets and contribute to “macrophage disappearance reaction” (3) while secreting high levels of inflammatory cytokines and chemokines that mediate interferon responses. This initial response recruits neutrophils (NEUs), monocytes, and complement components to the infection site. Monocyte-derived macrophages subsequently infiltrate the alveolar space, replenishing the resident macrophage pool and amplifying inflammation. However, excessive immune activation can lead to a dysregulated “cytokine storm,” marked by elevated chemokines, cytokines, and adhesion molecules, which exacerbates lung damage and significantly increases mortality in H5N1 infections (4). A detailed understanding of immune modulation during lung injury is essential to decipher the distinct pathogenesis of HPAIV compared with seasonal influenza.

Recent advances in single-cell RNA sequencing (scRNA-seq) and spatial transcriptomics now enable high-resolution dissection of cellular heterogeneity, intercellular communication, and tissue architecture. While these methods have been applied to study host responses to viruses such as SARS-CoV-2 (5), Rabies virus (6), and Epstein-Barr virus (7), they have also been used to profile influenza virus infection. Pioneering work by Kudo et al. used scRNA-seq to demonstrate that low ambient humidity impairs interferon-stimulated gene (ISG) expression across multiple cell types, thereby increasing susceptibility to severe disease (8). Other studies have further dissected the heterogeneity of immune cell responses to influenza virus infection at single-cell resolution (9). However, these studies have primarily focused on single virus strains (e.g., PR8) or environmental modifiers. To our knowledge, a direct comparative analysis of the immune landscapes elicited by seasonal H1N1 versus highly pathogenic avian influenza H5N1 (clade 2.3.2.1) using integrated scRNA-seq and spatial transcriptomics has not been reported. Here, we performed scRNA-seq on immune cells from murine lungs infected with H5N1 or H1N1 at multiple time points. Analysis of 84,162 cells identified 12 distinct immune phenotypes, including AMs, NEUs, monocytes, and T cells. By integrating these data with spatial transcriptomics, we mapped their distribution and temporal dynamics within the tissue. Through comparative analysis of viral pathogenicity, immune cell trafficking, and cytokine production, we delineate a distinct immunological landscape differentiating H5N1 from H1N1 infection.

## MATERIALS AND METHODS

### Mice and cells

Female BALB/c mice (6–8 weeks old, 18–22 g) were purchased from Beijing Sibeifu Biotechnology Co., Ltd. (License No. SCXK [Jing] 2024-0001). Mice were housed in specific pathogen-free (SPF) facilities under a 12-h light/dark cycle at 20°C–24°C and 60% ± 5% humidity. Madin-Darby canine kidney (MDCK) cells were obtained from South China Agricultural University. Murine alveolar macrophage (MH-S) cells were purchased from Cybio Biotechnology Co., Ltd. (Shanghai, China). MDCK cells and MH-S cells were cultured in DMEM and RPMI 1640 medium (Gibco, Grand Island, NE, USA), respectively. Both media were supplemented with 10% fetal bovine serum (FBS, Gibco), 100 U/mL penicillin, and 100 mg/mL streptomycin and maintained at 37°C in a 5% CO_2_ atmosphere.

### Virus titration

To model and compare the pathogenesis of a circulating, human-adapted influenza virus with that of a highly pathogenic avian influenza virus, we selected two representative strains. The pandemic-origin A/California/04/2009 (H1N1) virus (CA04) was kindly provided by Professor Xiaoquan Wang from Yangzhou University. The highly pathogenic avian influenza H5N1 virus used in this study was a clinical isolate obtained in our laboratory belonging to clade 2.3.2.1. Viruses were propagated in 9- to 11-day-old SPF chicken embryos. For TCID_50_ determination (10, 11), MDCK cells were seeded in 96-well plates and infected with 10-fold serial dilutions of virus. After 72 h of incubation at 37°C, wells were scored for cytopathic effect (CPE). The presence of the virus in CPE-positive wells was confirmed by hemagglutination assay. TCID_50_ titers were calculated using the Reed-Muench method. For EID_50_ determination (10, 11), 10-fold serial dilutions of virus were inoculated into the allantoic cavity of 9- to 11-day-old SPF embryonated chicken eggs (*n* = 3 eggs per dilution). After 48–72 h of incubation at 37°C, allantoic fluid was harvested and tested for hemagglutination activity. EID_50_ titers were calculated using the Reed-Muench method. In addition, mice were intranasally inoculated with serial virus dilutions (based on TCID_50_ values determined on MDCK cells: 7.54 log_10_ TCID_50_/mL for H1N1 and 7.24 log_10_ TCID_50_/mL for H5N1) and monitored for 14 days with daily body weight recording. Clinical signs included dyspnea, lethargy, reduced activity, hunched posture, poor coat condition, and diminished responsiveness. Mice losing ≥25% of the initial body weight were euthanized in compliance with welfare guidelines. The median lethal dose (MLD_50_) was calculated from mortality rates.

All experiments involving live H5N1 virus were conducted in a biosafety level 3 (BSL-3) laboratory at South China Agricultural University, following institutional biosafety protocols and in compliance with local and international biosafety regulations.

### Histopathology analysis

Lung tissues were fixed in 4% paraformaldehyde for a minimum of 48 h. Tissues were then dehydrated, paraffin-embedded, sectioned at 4 μm, and stained with hematoxylin and eosin (H&E). Stained sections were observed and imaged using a Nikon Eclipse E100 microscope equipped with a Nikon DS-U3 imaging system (Japan). To quantify pathological severity, a semi-quantitative lesion scoring system (range 0–5) was applied based on the assessment of inflammatory infiltration, structural disruption, and alveolar wall thickening. Scores were defined as follows: 0, absent; 1, minimal; 2, mild; 3, moderate; 4, marked; 5, severe. To eliminate observer bias, scoring was conducted under blinded conditions.

### Lung tissue single-cell suspension preparation

Mice were anesthetized, and lungs were perfused via the right ventricle with PBS. Tissues were dissected and digested in RPMI-1640 medium containing collagenase I, dispase II, and DNase I (Yeasen, Shanghai, China) at 37°C for 60 min (12). The digested tissue was filtered through a 70 μm strainer and centrifuged. Cells were resuspended in 40% Percoll (TBD, China), layered over 70% Percoll, and centrifuged. The interface layer (leukocytes) was collected, and red blood cells were lysed using ACK lysis buffer (TBD, Tianjin, China). After washing with PBS, the pellet (lung leukocyte fraction) was resuspended in sorting buffer. CD45^+^ cells were enriched using CD45 microbeads (Miltenyi, Bergisch Gladbach, Germany). Cell viability was assessed by AO/PI staining (Countstar, China) using a Countstar Rigel S3 system.

### Library preparation and sequencing

Single-cell RNA sequencing was conducted using the 10× Genomics Chromium platform (10× Genomics, Pleasanton, CA, USA) as previously described (13). Briefly, cell suspensions were adjusted to a concentration of 1,000–2,000 cells/μL and loaded onto a Chromium Controller to generate Gel Beads-in-Emulsions (GEMs) using the Chromium Single-Cell 3′ Reagent Kit and Gel Bead Kit according to the manufacturer’s instructions. After GEM generation, the gel beads were dissolved to release barcoded capture sequences, followed by reverse transcription to produce barcoded cDNA. The emulsion was then broken, and the cDNA was amplified by PCR. The products from all GEMs were pooled for standard library construction. For library preparation, the cDNA was fragmented to 200–300 bp, end-repaired and A-tailed, ligated with P5/P7 adapters and sample indexes, and finally PCR-amplified. The resulting libraries were subjected to high-throughput sequencing on an Illumina platform (Illumina, San Diego, CA, USA). All library preparation and sequencing procedures were performed by Genedenovo Biotechnology Co., Ltd. (Guangzhou, China).

Raw sequencing data were processed using Cell Ranger software (v5.0.0, 10× Genomics) for alignment, barcode processing, and UMI counting. Sequencing quality metrics for each sample are summarized in Table S1. Across all samples, the median number of reads was 467 million (range 376–647 million), and valid barcode rates ranged from 97.7% to 98.2%. Reads were mapped to the mouse reference genome GRCm39. Subsequent data analysis was performed using the Seurat package (v4.0) in R (14). Putative doublets were identified and removed using DoubletFinder (15). Low-quality cells were filtered based on the following criteria: (i) total UMI count per cell ≤34,000; (ii) percentage of mitochondrial genes ≤27%; and (iii) number of detected genes per cell between 200 and 5,000. Dimensionality reduction was carried out via principal component analysis (PCA). Batch effects were corrected using Harmony, and visualization was performed using t-distributed stochastic neighbor embedding (t-SNE) implemented in Seurat. Cell types were manually annotated based on canonical marker genes from the published literature (Table S2).

### Spatial transcriptome profiling

Spatial transcriptomic analysis of murine lung tissues was performed using the 10× Genomics Visium platform (with capture spots of 100 μm in diameter), following the manufacturer’s protocol. Briefly, lung samples were flash-frozen in isopentane, embedded in optimal cutting temperature (OCT) compound, and sectioned at 10 μm thickness using a cryostat. Tissue sections were mounted onto Visium Spatial Tissue Optimization Slides and Visium Spatial Gene Expression Slides. H&E staining was performed using reagents from Agilent (Santa Clara, CA, USA), and bright-field images were acquired using a Nikon microscope (Tokyo, Japan).

The Visium Spatial Tissue Optimization Slide kit (10× Genomics, PN-1000191) was used to determine the optimal permeabilization time, which was established as 12 min. Following permeabilization, tissue mRNAs were captured by spatially barcoded probes on the array, followed by reverse transcription, second-strand synthesis, PCR amplification, alkaline denaturation, fragmentation (200–300 bp), and library construction. Sequencing was performed on an Illumina platform following the 10× Genomics Visium workflow. Sequencing quality metrics are summarized in Table S3: across all samples, the median number of reads was 306.3 million (range 183.8–404.0 million), and valid barcode rates ranged from 97.80% to 98.71%. Data alignment and demultiplexing were performed using the Space Ranger pipeline. All subsequent analyses were carried out in R using the Seurat package (v4.0). The SCTransform algorithm was applied to normalize the data and account for sequencing depth differences, while the Harmony algorithm was used to correct for batch effects.

To integrate scRNA-seq and spatial transcriptomic data, we employed CellTrek (16) to map single cells to their spatial contexts within tissue sections. Briefly, both data sets are co-embedded into a shared low-dimensional feature space. A supervised random forest model is then trained to learn the relationship between gene expression and spatial location, ultimately predicting the most probable spatial coordinates for individual cells, thereby achieving single-cell spatial mapping.

### Differential gene expression and pathway analysis

Differential gene expression analysis between H5N1- and H1N1-infected groups for each cell type was conducted using the Seurat package. Differentially expressed genes (DEGs) were identified using the following thresholds: adjusted *P*-value ≤ 0.01, |log2FC|(fold change) ≥0.36, and detection in at least 25% of cells within a given cluster. Gene Ontology (GO) and Kyoto Encyclopedia of Genes and Genomes (KEGG) pathway enrichment analyses were performed on the identified DEGs. A protein-protein interaction (PPI) network related to cell death was constructed using the STRING database and visualized with Cytoscape (v3.9.1).

### Pseudotime and cell-cell communication analysis

Intercellular communication networks were inferred using CellChat (v2.1.2) (17) with the built-in mouse ligand-receptor database “CellChatDB.mouse.” Communication probabilities were computed at both signaling pathway and ligand-receptor levels. Significantly enriched pathways were visualized via heatmaps, and communication networks between immune cell subtypes under H5N1 and H1N1 infection conditions were constructed. The statistical significance (*P*-value) of each predicted ligand-receptor interaction was assessed through permutation testing, with interactions meeting the threshold of *P* ≤ 0.05 considered statistically robust.

Pseudotime trajectory analysis was performed using Monocle2 (v2.22.0) (18) in an unsupervised manner via reversed graph embedding. Cells were partitioned into subgroups through hierarchical clustering, and the biological functions of each subgroup were subsequently analyzed.

### *In vivo* cell depletion

To deplete alveolar macrophages, mice were intranasally administered 100 µL of clodronate liposomes (CL, Yesen, Shanghai, China) or an equivalent volume of PBS-containing liposomes as a control (11). The administration was performed twice: 48 h prior to influenza virus infection and 24 h post-infection. Survival rates were monitored throughout the experimental period.

For the depletion of pulmonary monocytes and NEUs, mice received an intraperitoneal injection of 150 µg anti-Ly6G rat monoclonal antibody (Clone 1A8) or anti-Ly6C rat monoclonal antibody (Clone Monts 1) 24 h before and 48 h after viral infection (19). Control animals were administered an equivalent dose of IgG2a isotype control antibody (Clone 2A3). All antibodies were procured from BioXCell (Lebanon, NH, USA). The efficiency of depletion for each cell type was assessed using flow cytometry.

### Flow cytometry

Isolated leukocytes were incubated with an anti-CD16/32 antibody (Clone 93) to block non-specific binding, following the manufacturer’s instructions. Cells were then stained with a cocktail of fluorescently conjugated antibodies, including CD45-Brilliant Violet 510 (Clone 30-F11, 0.5 μg per test), CD170-PE Siglec-F (Clone S17007L, 0.25 μg per test), CD3-APC/Cyanine7 (Clone 17A2, 0.25 μg per test), CD11b-PerCP/Cyanine5.5 (Clone M1/70, 0.25 μg per test), CD11c-APC (Clone N418, 0.25 μg per test), Ly6G-PE/Cyanine7 (Clone 1A8, 0.25 μg per test), and Ly6C-FITC (Clone HK1.4, 0.25 μg per test). All antibodies were obtained from BioLegend (San Diego, CA, USA). Apoptosis and necrosis in murine AMs were quantified using an Annexin V-FITC/PI detection kit (Solarbio, Beijing, China). Harvested cells were washed in cold PBS and resuspended in 100 µL of 1× binding buffer. The suspension was stained with 5 µL of Annexin V-FITC and PI for 5 min at room temperature in the dark. Data acquisition was performed on a CytoFLEX flow cytometer (Beckman Coulter, Brea, CA, USA), and analysis was conducted using FlowJo software (v10.8.1, Tree Star Inc., Ashland, OR, USA) (12).

### Viral internalization assay

A total of 150,000 MH-S cells were seeded in flat-bottom confocal dishes in 1 mL RPMI 1640 medium per well and incubated at 37°C with 5% CO_2_ for 16–18 h. To stain the cytoplasm, 1 μM CellTracker Green CMFDA (Beyotime, Shanghai, China) was added, and cells were washed 2× with medium to remove excess dye. H1N1 and H5N1 virions were separately labeled with 2 μM pHrodo sulfotetrafluorophenyl (STP) ester (Thermo Fisher Scientific, Waltham, MA, USA) for 40 min at 37°C. The labeled virions were then purified by ultracentrifugation to remove unbound dye, resuspended in HBSS, and added to MH-S cells. After 1.5 h of co-incubation, cells were washed twice with HBSS. Finally, cells were imaged using a confocal laser scanning microscope (Olympus, Japan).

### Statistical analysis

Statistical analyses were carried out using GraphPad Prism (v10.5, GraphPad Software Inc., San Diego, CA, USA). Survival curves were compared using the log-rank test. Data are expressed as mean ± SD. For comparisons between two groups, an unpaired t-test was used. For comparisons involving three or more groups, a one-way ANOVA was conducted to determine overall significance; if significant, pairwise multiple comparisons were performed using Tukey’s test. A *P*-value of less than 0.05 was considered statistically significant (*P < 0.05, **P < 0.01, ***P < 0.001).

## RESULTS

### Distinct replication and pathogenesis of H1N1 and H5N1 influenza viruses in mice

**Fig 1:**
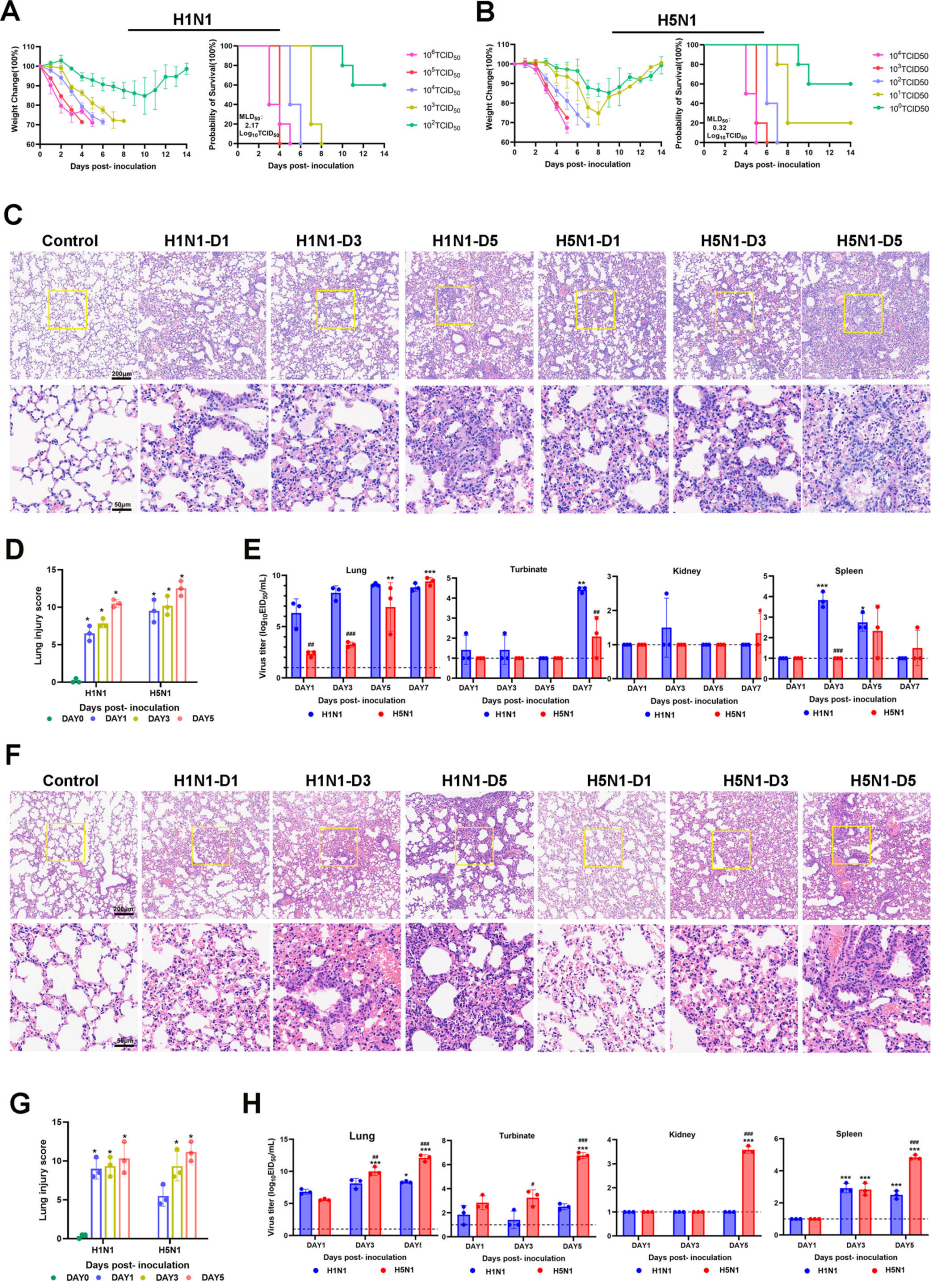
Pathogenesis and replication of H5N1 and H1N1 influenza viruses in mice. (**A and B**) BALB/c mice (*n* = 5/group) were intranasally inoculated with 10-fold serial dilutions of H1N1 or H5N1 virus. Body weight change (A, left) and survival (A, right) were monitored daily for 14 days. Mice losing ≥25% of the initial body weight were humanely euthanized. The MLD_50_ was calculated using the Reed-Muench method (**B**). (**C**) Representative H&E-stained lung sections from mice infected with 100 TCID_50_ of H1N1 or H5N1 at 1, 3, and 5 days post-infection (dpi; *n* = 3/group). Lower panels show higher magnification of the boxed regions in the upper panels. (**D**) Histopathological scores for inflammation, structural disruption, and alveolar wall thickening in the sections from panel C. Scores (0–5) are based on published criteria. Data are mean ± SD (*n* = 3/group). (**E**) Viral titers in lung, spleen, nasal turbinate, and kidney tissues from mice in panel C were determined by EID_50_ assay in embryonated chicken eggs. Data are mean ± SD (*n* = 3/group). (**F–H**) Experiments in panels **C–E** were repeated in mice infected with 1× MLD_50_ of H1N1 or H5N1. (**F**) Representative H&E-stained lung sections (*n* = 3/group). (**G**) Corresponding histopathological scores (mean ± SD). (**H**) Viral titers in tissues from mice in panel F (mean ± SD). Significance is indicated as follows: *Control vs. IAV; #H1N1 vs. H5N1.

To systematically compare the pathogenicity of seasonal and highly pathogenic avian influenza viruses in mammals, we used a well-established mouse model. Mice were intranasally inoculated with serial dilutions of H1N1 or H5N1 virus and monitored daily for 14 days. At doses normalized by TCID_50_ on MDCK cells (10^4^ TCID_50_), H5N1-infected mice exhibited more rapid weight loss and earlier mortality than H1N1-infected mice. The MLD_50_ of H5N1 was calculated to be 0.32 log_10_ TCID_50_, significantly lower than that of H1N1 (2.17 log_10_ TCID_50_) (Fig. 1A and B).

Histopathological analysis of lungs from mice infected with 100 TCID_50_ revealed that H5N1 induced more severe and rapidly progressive pulmonary pathology than H1N1. Although histological scores were comparable at 1 dpi, H5N1-infected mice showed markedly elevated lung injury scores by 3 dpi, with diffuse alveolar damage, alveolar wall thickening, cellular death, dense inflammatory infiltrates, and disrupted parenchymal architecture (Fig. 1C and D).

Viral replication kinetics differed substantially between the two viruses (Fig. 1E). While H1N1 lung titers were higher at 1 dpi, H5N1 titers significantly surpassed H1N1 by days 3 and 5. H5N1 also exhibited broader and earlier extra-pulmonary dissemination. In nasal turbinates, H5N1 detection rates and titers exceeded those of H1N1 on days 3 and 5. In the spleen, H5N1 replication was significantly higher than H1N1 on day 5. Moreover, H5N1 was detected in kidney tissues on day 5, whereas H1N1 was not found in the kidneys at any time point. These findings suggest that while H1N1 establishes initial infection more readily in the respiratory tract, the severe lethality of H5N1 stems from enhanced delayed replication and efficient systemic dissemination to extra-respiratory organs, rather than higher initial infectivity.

To investigate pathogenic mechanisms under conditions of equivalent lethality, we challenged mice with 1× MLD_50_ of each virus. Histopathological analysis showed that H1N1-infected mice exhibited more severe lung injury than H5N1-infected mice at 1 dpi, but injury scores were comparable by days 3 and 5 (Fig. 1F and G). Viral replication kinetics also diverged (Fig. 1H). In lungs, H1N1 titers remained relatively stable throughout infection, whereas H5N1 titers increased progressively. Although H5N1 titers were significantly lower than H1N1 on days 1 and 3, they reached comparable levels by day 5. In nasal turbinates, H1N1 was sporadically detected on days 1 and 3, while H5N1 became detectable only on day 7, with titers remaining significantly lower than H1N1. In the spleen, H1N1 replication peaked on day 3 and declined to near-undetectable levels on day 7, consistent with viral clearance. In contrast, H5N1 titers in the spleen remained low throughout. No virus was detected in the kidneys of either group.

Notably, these results contrasted sharply with the robust replication and systemic spread of H5N1 observed in the high-dose (100 TCID_50_) challenge. Under lethal-dose conditions, H5N1 did not demonstrate enhanced virulence or replicative fitness; instead, its replication kinetics and pathological progression were delayed or attenuated. The exceptionally high lethality of highly pathogenic avian influenza virus, reflected in its extremely low LD_50_, stems primarily from its ability to rapidly breach host defenses upon high-dose exposure, establish high viral load, and achieve systemic dissemination, leading to fatal outcomes. By contrast, the seasonal influenza virus establishes early infection more efficiently and replicates in a manner that better preserves host homeostasis. Consequently, its pathogenicity arises primarily from immunopathological responses rather than direct virus-mediated cytolysis.

### Single-cell profiling of immune responses to H1N1 and H5N1 influenza infections in murine lungs

**Fig 2:**
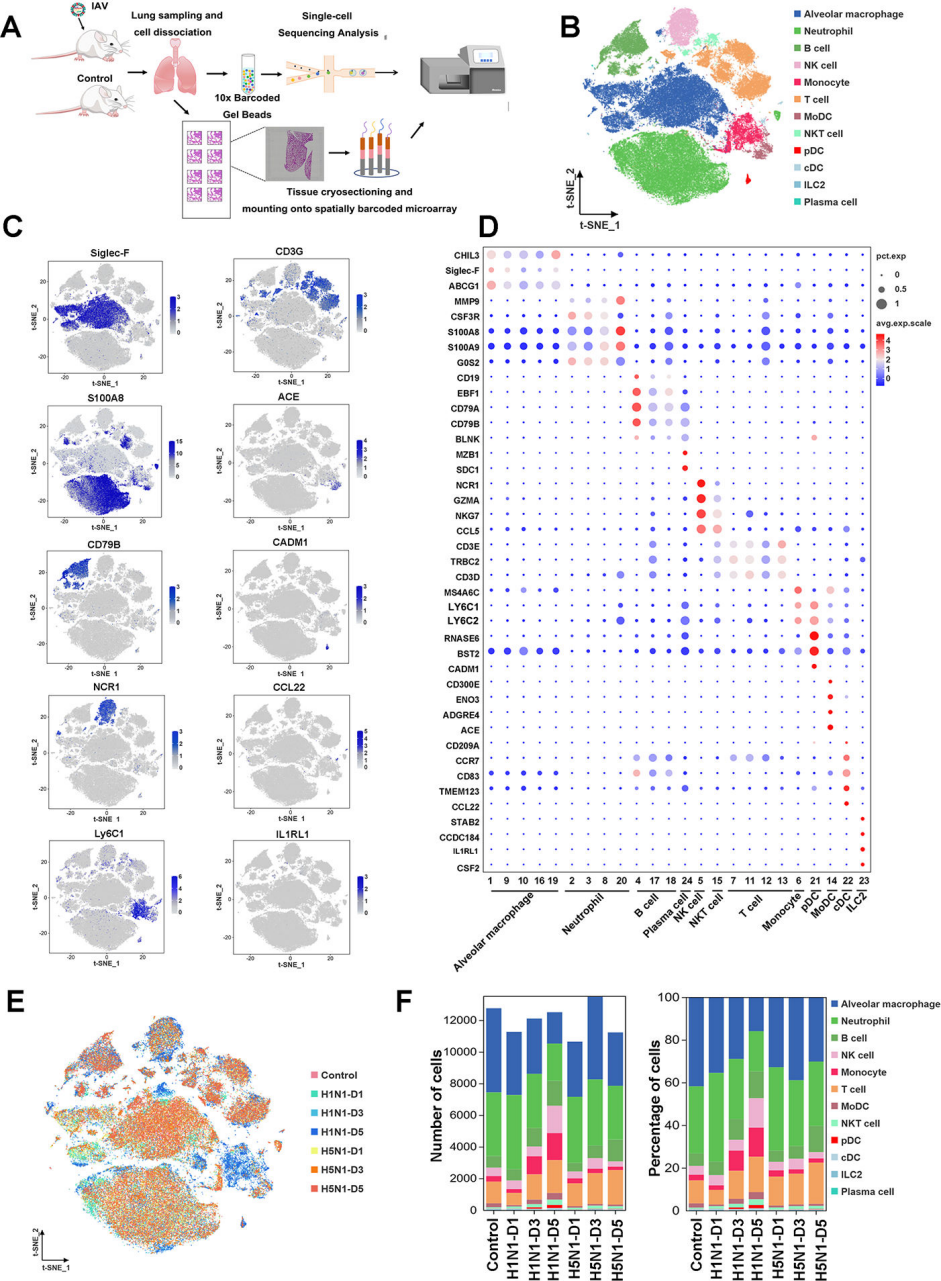
Single-cell and spatial transcriptomic atlas of the murine lung immune landscape following influenza virus infection. (**A**) Experimental workflow. Single-cell suspensions were generated from lungs of control, H1N1-infected (1× MLD_50_), and H5N1-infected mice at 1, 3, and 5 dpi. For scRNA-seq, cells from three biological replicates per condition were pooled. Consecutive lung sections from the same cohorts were used for spatial transcriptomics. (**B**) t-SNE plot of all cells (*n* = 84,162) from control and infected lungs, colored by 12 immune cell types identified by canonical marker expression. (**C**) Feature plots showing expression patterns of key marker genes used for cell type annotation. (**D**) Dot plot summarizing canonical marker expression for each cell cluster. Dot size indicates the percentage of expressing cells; color intensity indicates the average expression level. (**E**) t-SNE plots showing the transcriptomes of all cells, stratified by sample and infection condition. (**F**) Stacked bar graphs showing absolute cell counts (left) and proportional abundance (right) of each immune cell type across all samples.

To compare immune responses elicited by seasonal versus highly pathogenic avian influenza virus under conditions of equivalent lethality, we performed scRNA-seq on immune cells isolated from mouse lungs at 1, 3, and 5 days after intranasal inoculation with 1× MLD_50_ of H1N1 or H5N1 (Fig. 2A). After quality control (Fig. S1A), we obtained a high-quality data set of 84,162 cells (Tables S1 and S4). Unsupervised clustering via t-SNE identified 24 distinct cell clusters (Fig. S1B), which were annotated into 12 immune cell types based on canonical marker gene expression (Fig. 2B through D; Table S5): AMs, NEUs, NK cells, NKT cells, monocytes, B cells, plasma cells, T cells, myeloid dendritic cells (MoDCs), plasmacytoid dendritic cells (pDCs), conventional dendritic cells (cDCs), and type 2 innate lymphoid cells (ILC2s).

Myeloid populations underwent substantial temporal remodeling during infection. Compared to uninfected controls, H1N1 infection induced the classical “macrophage disappearance reaction,” which intensified over time, along with progressive monocyte enrichment and a neutrophil response that increased before decreasing. In contrast, H5N1 infection caused an initial reduction followed by a rebound in AMs, accompanied by modest monocyte recruitment and sustained neutrophil infiltration. MoDCs increased over time after H1N1 infection but decreased after H5N1 infection, while other dendritic cell subsets expanded in response to both viruses. Lymphoid populations (B, NK, and T cells) also exhibited time-dependent changes (Fig. 2E and F; Fig. S1C). Reads were mapped to the reference genomes of the H1N1 and H5N1 strains used. Low levels of viral reads were detected in alveolar macrophages, monocytes, and neutrophils, consistent with the remodeling of myeloid cells after infection (Fig. S1D).

Global transcriptional comparisons revealed that H5N1 infection elicited fewer differentially expressed genes than H1N1 (Fig. S1E and F). GO enrichment analysis showed a temporal progression of immune processes: cell death and metabolism pathways were enriched at 1 dpi, immune response and defense terms at 3 dpi, and adaptive immunity activation by 5 dpi. H5N1 infection uniquely enriched terms related to oxidative stress and lipid metabolism (Fig. S1G), indicating distinct host response mechanisms.

### Spatial mapping of single-cell transcriptomes in murine lungs upon H1N1 and H5N1 infection

**Fig 3:**
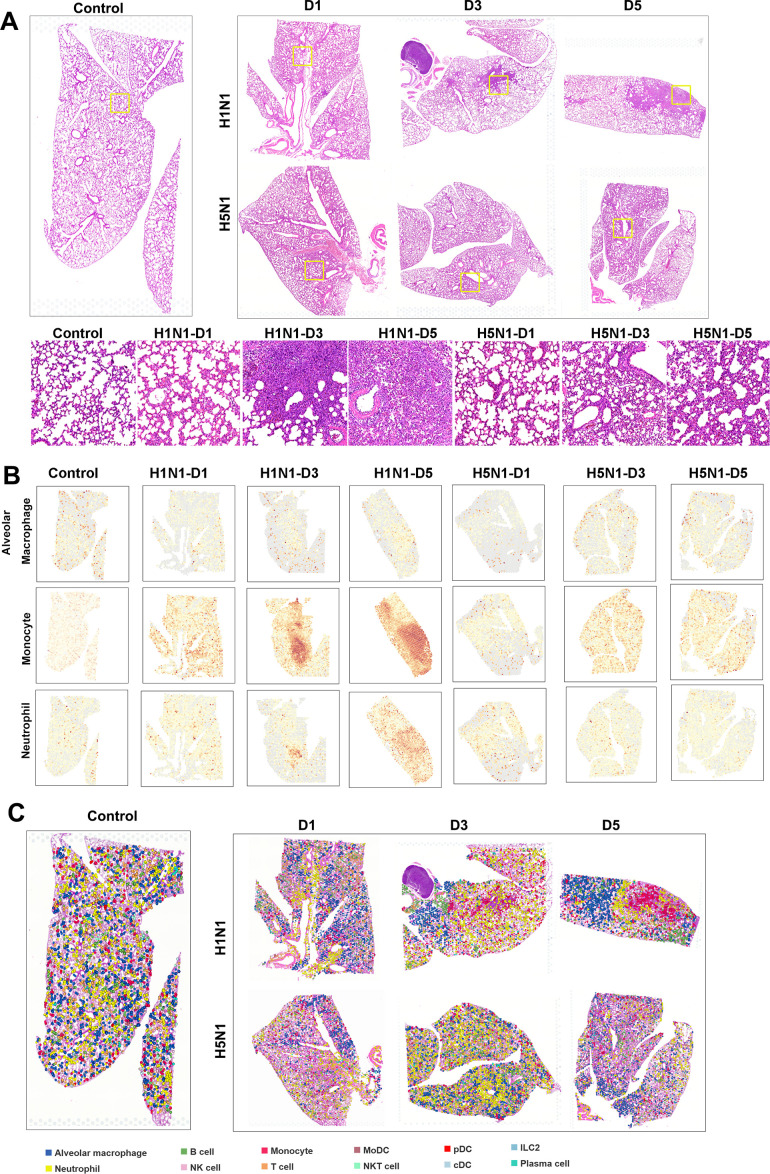
Spatial transcriptomic atlas of murine lung tissues pre- and post-infection with H1N1 and H5N1 influenza viruses. (**A**) Representative H&E-stained lung sections from control, H1N1-infected, and H5N1-infected mice. Upper panels show whole-tissue overviews; lower panels show magnified views of the boxed regions. (**B**) Spatial distribution of canonical marker genes for AMs (CHIL3^+^Siglec-F^+^ITGAX^+^ABCG1^+^), neutrophils (MMP9^+^CSF3R^+^S100A8^+^S100A9^+^G0S2^+^), and monocytes (Ly6C1^+^Ly6C2^+^CCR2^+^MS4A6C^+^), as identified by CellTrek. (**C**) Integration of scRNA-seq and spatial transcriptomics data via CellTrek, mapping the 12 annotated immune cell types to their spatial coordinates within lung tissue sections.

To investigate spatial alterations in immune cell distribution during peak infection periods of seasonal IAV and highly pathogenic avian influenza virus, we conducted unbiased spatial transcriptomic analysis of H1N1- and H5N1-infected lung tissues using the 10× Genomics Visium platform (Fig. 2A). This approach allowed us to first assess the pathological changes following H1N1 and H5N1 infection, thereby better defining the spatial alterations in immune cells within the damaged lung areas. Consistent with the quantitative histological scoring from three mice per time point (Fig. 1G), pathological assessment of the spatial transcriptome sections revealed a time-dependent increase in damaged tissue, with the most extensive changes observed at 5 dpi (Fig. 3A). Influenza virus-infected mice exhibited characteristic pathological features, including disrupted alveolar architecture, markedly thickened alveolar walls, inflammatory cell infiltration within the interstitial compartment, and evident vascular dilation and congestion. H1N1 infection produced bronchocentric dissemination of pathological changes, resulting in focal lesions, whereas H5N1 infection induced progressive and diffuse pulmonary damage. We visualized the spatial expression patterns of marker genes for AMs (CHIL3^+^Siglec-F^+^ITGAX^+^ ABCG1), monocytes (Ly6C1^+^Ly6C2^+^CCR2^+^MS4A6C), and neutrophils (MMP9^+^ CSF3R^+^S100A8^+^S100A9^+^G0S2) in control, H1N1-infected, and H5N1-infected groups using the SpatialFeaturePlot function in Seurat (Fig. 3B). Macrophage marker gene expression significantly increased during later infection stages (days 3 and 5), suggesting a critical role for these cells in the early phase of infection. Monocyte marker genes were more highly upregulated in H1N1-infected than in H5N1-infected mice, indicating restricted monocyte activation in H5N1 infection. Neutrophil marker genes were significantly upregulated as early as 1 dpi, indicative of an activated neutrophil phenotype during the initial stage of the host response.

To resolve spatial heterogeneity in the dynamics of these myeloid populations, we integrated spatial transcriptomics with scRNA-seq, mapping annotated immune cells to specific tissue coordinates (Fig. 3C). This integrative approach revealed distinct temporal dynamics of immune cell recruitment. In H1N1-infected lungs, AMs declined progressively over time, while recruited monocytes accumulated markedly at lesion sites, consistent with their potential to differentiate into macrophages and repopulate the depleted resident pool. Neutrophils underwent transvascular migration (observable at 1 dpi) and accumulated at lesion sites by days 3 and 5. In contrast, H5N1 infection elicited a more modest reduction in AMs and a less pronounced increase in monocytes, but triggered a significantly stronger neutrophil response, with substantial infiltration particularly evident at 3 dpi (Fig. 3C).

In summary, integration of spatial transcriptomics and scRNA-seq data validated distinct responses of alveolar macrophages, monocytes, and neutrophils to H1N1 and H5N1 infection.

### Alveolar macrophage cell death and the early myeloid response in H1N1 and H5N1 infection

**Fig 4:**
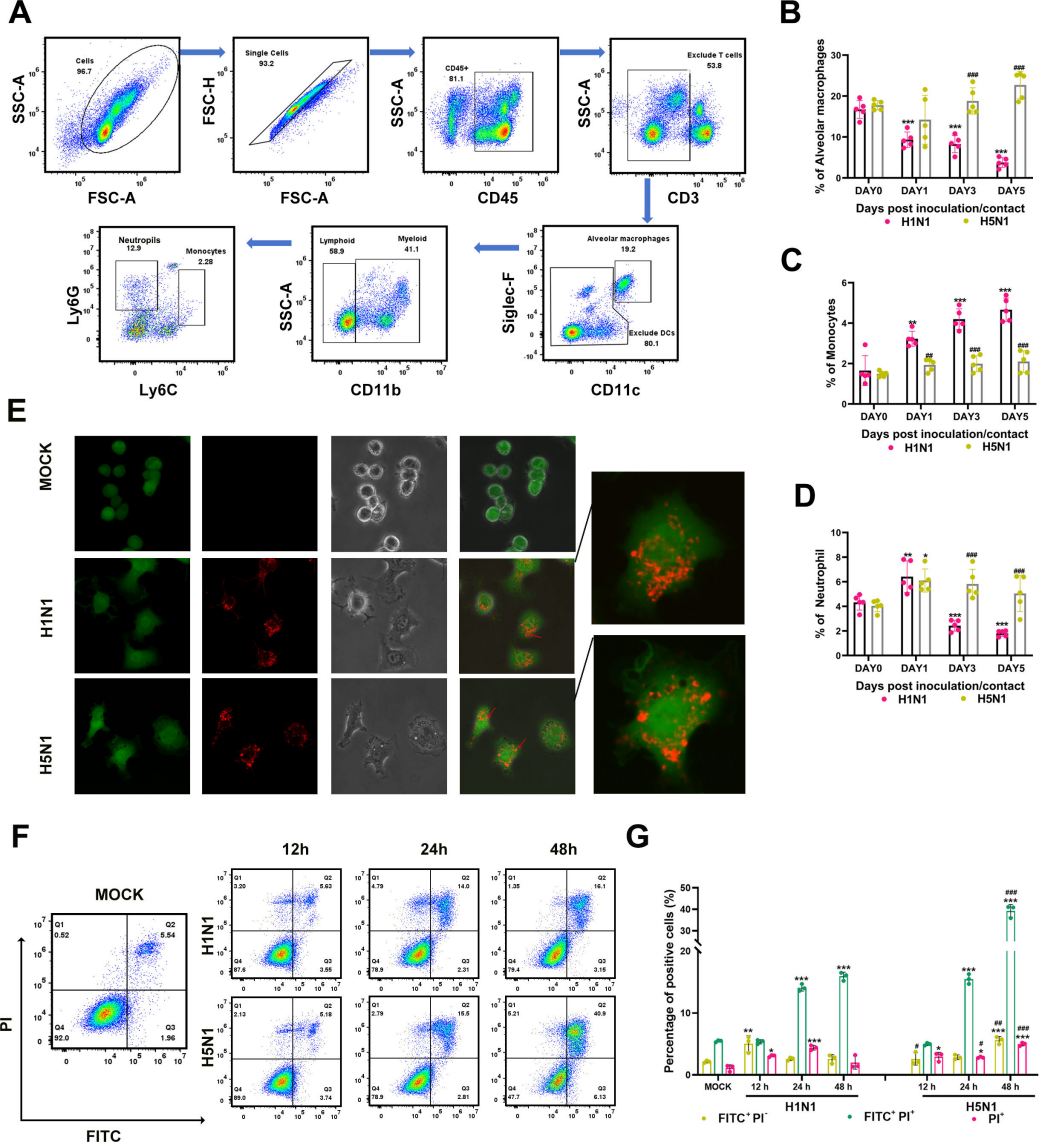
Macrophage internalization and death kinetics in H1N1 and H5N1 infection. (**A**) Gating strategy for identifying myeloid populations: debris and doublets were excluded using FSC-A/SSC-A and FSC-H parameters; CD45^+^ immune cells were selected, followed by exclusion of CD3^+^ lymphocytes. Alveolar macrophages (CD11c^+^ Siglec-F^+^), monocytes (CD11b^+^ Ly6C^+^), and neutrophils (CD11b^+^ Ly6G^+^) were subsequently gated. (**B–D**) Quantitative analysis of alveolar macrophages (**B**), monocytes (**C**), and neutrophils (**D**) in control and infected groups (*n* = 5/group). (**E**) Confocal imaging of MH-S cells (cytoplasm labeled with CMFDA, green) after 1.5-h incubation with pHrodo-labeled H1N1 or H5N1 virus (red). Red fluorescence indicates viral uptake into acidic phagolysosomes. Scale bar, 20 μm. (**F and G**) Flow cytometric analysis of apoptosis and necrosis in MH-S cells infected with H1N1 or H5N1 (MOI = 5) for the indicated times. (**F**) Representative plots. (**G**) Quantification of apoptotic (Annexin V^+^) and necrotic (PI^+^) cells. Data are mean ± SD (*n* = 3 independent experiments). Significance: *Control vs. IAV; #H1N1 vs. H5N1.

To validate these findings, we performed flow cytometry on immune cells isolated from infected lung tissues (Fig. 4A). The proportional changes in myeloid subsets were largely consistent with scRNA-seq frequencies and spatial distributions. After H1N1 infection, CD11c^+^Siglec-F^+^ AMs declined over time, while CD11b^+^Ly6C^+^ monocytes increased progressively, and CD11b^+^Ly6G^+^ neutrophils showed an initial increase followed by a decrease (Fig. 4B through D). Following H5N1 infection, AMs exhibited an initial decrease followed by a rebound, alongside a modest increase in monocytes. Neutrophil counts also rose and then declined, though overall cellular dynamics were more constrained than in H1N1 infection. Neutrophil dynamics measured by flow cytometry and spatial mapping showed modest quantitative divergence, but both data sets consistently captured an initial increase followed by a decrease. Spatially, neutrophils were initially confined to perivascular regions and progressively redistributed to discrete lesion cores, where they formed clusters—consistent with focal effector function at infectious foci.

To directly assess whether viral internalization is associated with AM death, we developed a fluorescence-based assay using pHrodo-labeled viruses and CMFDA-stained AMs. Both H1N1 and H5N1 viruses were rapidly internalized by AMs and trafficked to phagolysosomes, where acidification intensified pHrodo fluorescence, indicating efficient internalization and lysosomal delivery (Fig. 4E). Flow cytometry revealed that both late apoptosis (FITC^+^PI^+/−^) and necrosis (FITC^−^PI^+^) increased with higher viral MOI and longer infection time. Virus-associated cell death was more pronounced for H5N1, leading to substantially higher total mortality under high-dose, long-duration conditions (Fig. S2, S4F, and G). These findings demonstrate that early during infection, both H1N1 and H5N1 are associated with AM death, an event consistent with viral internalization via phagocytosis, and that this cellular demise correlates with rapid AM reduction and subsequent monocyte and neutrophil recruitment to lung injury sites.

### Interferon and inflammatory responses in myeloid cells during peak H1N1 and H5N1 infection

To systematically assess cellular responsiveness to interferons and inflammatory signals, we computed an IFN score and an inflammatory score for each immune cell subset by evaluating the expression profiles of ISGs and inflammatory response genes, respectively (Table S6). These scores were generated using the AddModuleScore function in Seurat. Differences in scores across all cell subtypes were assessed using the Kruskal-Wallis test.

**Fig 5:**
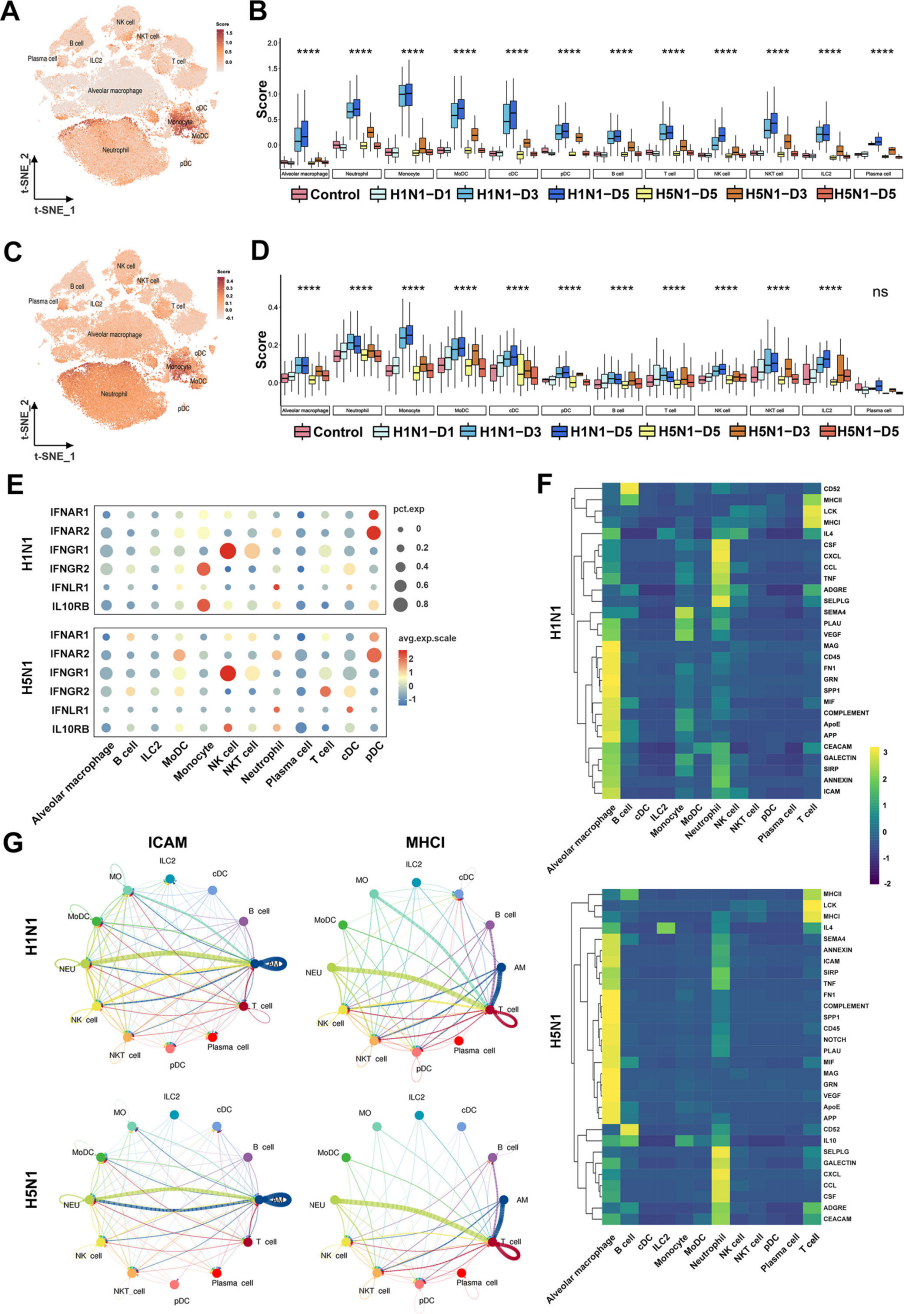
Interferon, inflammatory response, and signaling responses in murine lungs infected with H1N1 and H5N1. (**A and C**) t-SNE plots visualizing the interferon response score (**A**) and inflammatory response score (**C**) for the 12 identified immune cell types across the control, H1N1-infected, and H5N1-infected groups. (**B and D**) Bar plots showing the IFN and inflammatory response scores across all immune cell types, reflecting differential cellular responsiveness to interferon and inflammatory signals between H1N1 and H5N1 infection. Statistical significance among groups was assessed by the Kruskal-Wallis H test; a *P* value < 0.05 was considered significant. (**E**) Expression levels of type I (IFNAR1 and IFNAR2), type II (IFNGR1), and type III (IFNLR1) interferon receptor subunits, as well as the shared subunit IL10RB, across all identified cell types. (**F**) CellChat analysis depicting the outgoing and incoming signaling pathways among all cell types in the H1N1- and H5N1-infected groups. (**G**) Visualization of ICAM and MHCI signaling pathways in all cell types within the H1N1 and H5N1 infection groups. The size of each outer circle is proportional to the number of ligand–receptor pairs per cell group. Arrows indicate the direction of communication (from sender to receiver cells). Edge width represents the communication probability.

We first established an IFN score for each cell in our scRNA-seq data set based on the expression of 102 ISGs (20) (Fig. 5A). All 12 identified immune cell types, encompassing both myeloid and lymphoid lineages, exhibited significantly elevated IFN scores, with myeloid cells (especially monocytes) demonstrating the highest levels (Fig. 5B). We subsequently derived an inflammatory score (21) from the expression of 218 inflammatory response genes (Fig. 5C). Inflammatory scores were significantly elevated across all cell types except plasma cells. Temporal analysis revealed that both IFN and inflammatory scores increased progressively over time in H1N1-infected clusters. In contrast, H5N1-infected clusters reached peak scores at 3 dpi, with overall lower magnitudes compared to H1N1 (Fig. 5D).

We further analyzed the expression of ISGs and inflammatory factors in key myeloid populations, including alveolar macrophages, monocytes, and neutrophils. Numerous canonical ISGs, including ISG15, MX1, IFITM1, IFITM3, and IRF7, were significantly upregulated during peak IAV infection relative to controls. However, their expression levels were markedly lower in H5N1 infection compared to H1N1 (Fig. S3A). Similarly, inflammatory scores indicated that H1N1 infection induced substantial upregulation of multiple inflammatory factors, whereas H5N1 elicited a comparatively weaker response (Fig. S3B).

To evaluate potential mechanisms underlying these differential responses, we examined the expression of IFN receptors across cell types. IFN-α receptor subunits IFNAR1 and IFNAR2 were nearly ubiquitously expressed. In contrast, IFNGR1 (IFN-γ receptor 1) showed low expression in neutrophils, and IFNLR1 (IFN-λ receptor 1) was generally low across all cell types. Conversely, IL10RB, which forms a heterodimeric receptor complex with IFNLR1, was widely expressed (Fig. 5E; Fig. S3C). Collectively, these data suggest that, in this murine model of lethal-dose infection with a clade 2.3.2.1 H5N1 virus, the classical “cytokine storm” may not be the primary driver of pathogenesis, as the overall interferon and inflammatory responses were lower in H5N1-infected mice compared to H1N1.

Given the critical role of intercellular communication in regulating immune homeostasis and disease progression, we utilized CellChat to characterize cytokine- and membrane protein-mediated interactions during H1N1 and H5N1 infection. Relative to uninfected controls, H1N1 infection exhibited a greater number of ligand-receptor pairs and stronger communication strength than H5N1 infection (Fig. S3D). Among myeloid cells, AMs, monocytes, and neutrophils demonstrated heightened incoming and outgoing signaling activity (Fig. 5F). We specifically visualized the ICAM1 signaling pathway, which is essential for leukocyte trafficking and recruitment, and observed significantly reduced signaling in H5N1-infected groups compared to H1N1-infected groups.

Additionally, we assessed the MHCI signaling pathway, which is critical for CD8 T-cell recognition and cytotoxic response initiation. This pathway mediated weaker intercellular communication in H5N1 infection relative to H1N1 infection (Fig. 5G). These findings suggest that myeloid cells exhibit heightened signaling activity during acute lung injury. Additionally, H5N1 infection is associated with reduced ICAM1 and MHC-I signaling activity, which may correlate with altered immune cell recruitment and adaptive immune activation.

### Alveolar macrophage depletion and the course of pulmonary infection

AMs serve as sentinel immune cells during IAV infection and are critical for maintaining pulmonary surfactant homeostasis, defending against pathogens, and modulating immune responses. To investigate their role in severe pulmonary infection, we analyzed 26,901 AMs from mice infected with either H1N1 or H5N1 influenza viruses, which exhibit differing pathogenicity. Substantial remodeling of the AM population was observed in infected lungs compared to healthy controls (Fig. S1C).

**Fig 6:**
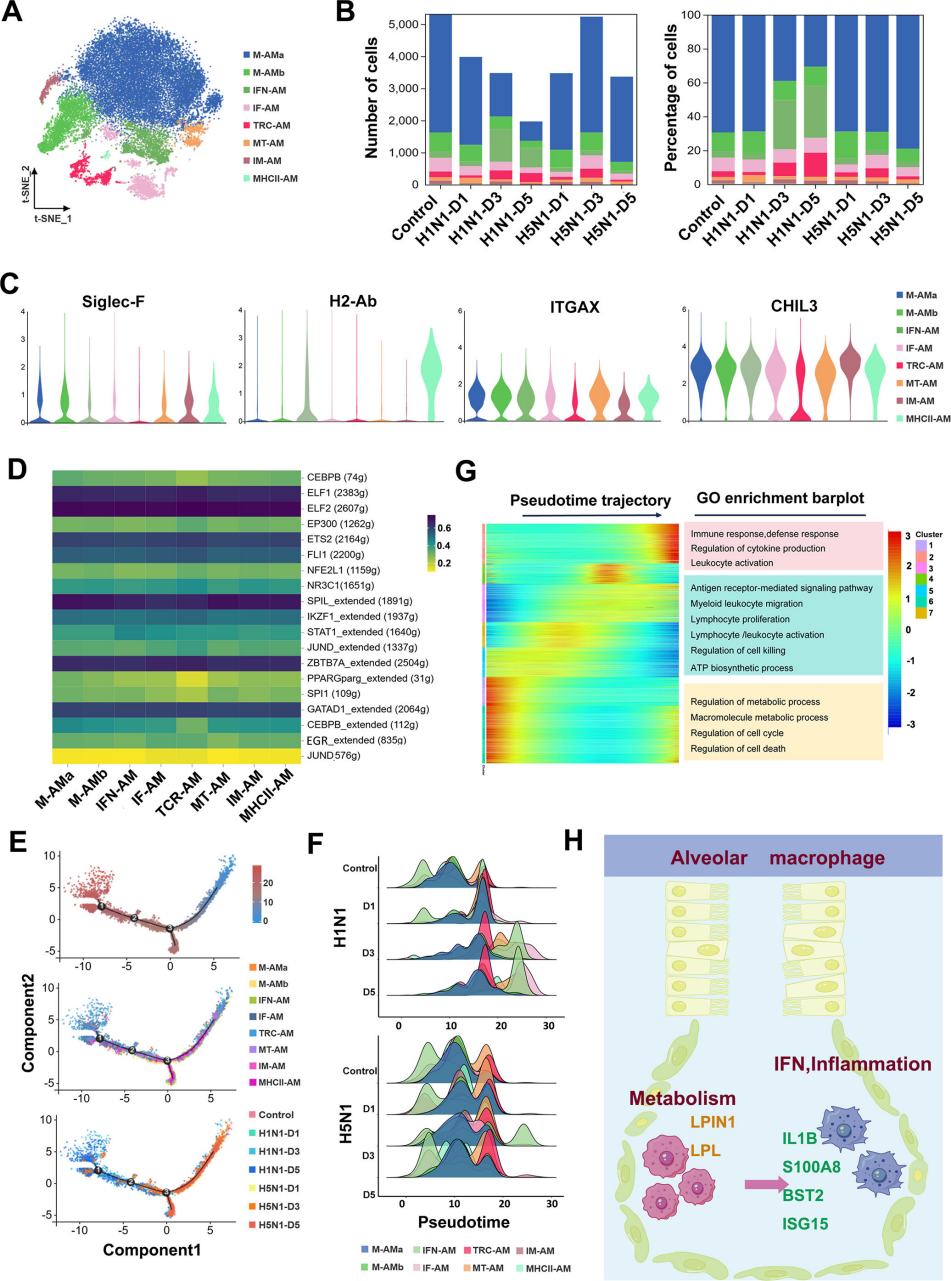
Immunophenotypic and functional characterization of alveolar macrophages in the IAV-infected murine lungs. (**A**) t-SNE visualization of reclustered AMs from control, H1N1-, and H5N1-infected groups, revealing eight subclusters. (**B**) Absolute counts (left) and proportional abundance (right) of AM subclusters per sample. (**C**) Dot plot showing expression of canonical marker genes for AM subcluster annotation. (**D**) Heatmap depicting transcription factor (TF) regulon activity across AM subclusters. (**E**) Pseudotime trajectory analysis of AM differentiation. Each point represents a single cell, colored by pseudotime (top), subcluster (middle), and sample origin (bottom). Branch points are numbered. (**F**) Ridge plots showing the abundance dynamics of AM subclusters along pseudotime for each sample group. (**G**) GO biological process terms enriched for genes differentially expressed at key trajectory branch points. (**H**) Schematic model summarizing the transition from a metabolic to an inflammatory transcriptional signature during AM differentiation in response to influenza infection.

To further characterize AM heterogeneity, we performed reclustering analysis (Fig. 6A). Based on established classification criteria and canonical marker genes, AMs were categorized into seven distinct subpopulations (Fig. 6B and C; Fig. S4A). Cluster 7 exhibited high expression of MHC class II genes (H2-Aa, H2-Ea, and H2-Eb1), indicative of antigen-presenting capability, and was designated MHCII-AM. Cluster 5 demonstrated elevated expression of metallothioneins and metal transporters, implicated in metal ion homeostasis, oxidative stress mitigation, and inflammatory responses (22), and was annotated as MT-AM. Clusters 0/1, 2, and 3 were defined by high expression of metabolic genes, ISGs, and inflammatory genes, respectively, and were labeled M-AMa/b, IFN-AM, and IF-AM. Cluster 4 displayed a unique transcriptional profile featuring T-cell receptor (TCR) components such as LAT and LCK. Given that TCR-positive macrophages have been reported primarily in inflammatory and infectious contexts (23, 24), this subset was designated TCR-AM. To exclude misannotation or macrophage-T-cell doublets, we confirmed that these cells expressed canonical AM markers (CHIL3 and ITGAX) at higher proportions than T-cell markers (CD3) and formed a distinct cluster separate from other subsets, with no outliers in quality metrics (Fig. S5A through D; Table S7).

Regulon activity analysis, which evaluates transcription factors and their target genes, revealed altered signaling pathways across AM subsets during infection. All subsets exhibited high activity of ETS family TFs, which regulate proliferation, differentiation, and apoptosis. Additionally, subsets other than those with metabolic functions showed elevated STAT1 and JUND activity. TCR-AM diverged notably from conventional AMs, displaying reduced activity of lipid metabolism regulators CEBPB and PPARG (Fig. 6D).

Pseudotemporal trajectory analysis identified two differentiation pathways originating from proliferating AMs and terminating in either antiviral inflammatory subsets or TCR subsets (Fig. 6E). Ridge plot visualization indicated that H1N1 infection promoted differentiation into later stages, whereas H5N1 infection resulted in accumulation of early-differentiated AMs (Fig. 6F). Notably, H5N1-infected mice showed predominant enrichment of metallothionein-expressing AMs, a phenotype recently associated with the early exudative phase of diffuse alveolar damage in COVID-19 (25).

GO enrichment analysis of pseudotime-dependent DEGs revealed that early-expressed genes in Clusters 1 and 6 were involved in metabolic processes and cell death regulation (Fig. 6G). Terminal trajectory genes in Cluster 2 were enriched in immune response and defense pathways, particularly cytokine production. Mid-trajectory genes in Clusters 3, 4, 5, and 7 were associated with antigen processing and presentation, lymphocyte activation, and cell-mediated cytotoxicity. Time-resolved lineage tracing demonstrated downregulation of metabolic genes (LPL and LPIN1) and upregulation of inflammatory and ISG genes (IL1B, S100A8, ISG15, and BST2) along the pseudotemporal axis, indicating a shift from metabolic activity toward an antiviral inflammatory state (Fig. 6H; Fig. S4B).

These findings suggest that H5N1 infection is associated with impaired AM activation and reduced antiviral functionality, which may contribute to a state of reduced immune responsiveness. Comparative DEG analysis between H1N1- and H5N1-infected AMs revealed 752 upregulated genes in H1N1 infection versus 274 in H5N1 infection relative to controls (Fig. S1D and E). Although both infections elevated expression of monocyte/neutrophil chemokines (CCL2, CCL3, CCL4, CCL5, CCL7, CXCL1, CXCL2, and CXCL3) (26) and ICAM1, these increases were attenuated in H5N1 infection (Fig. S3B). Conversely, H5N1 infection resulted in higher expression of cytokine storm mediators (IL1A, IL1B, and IL18) (27, 28), suggesting that AMs may contribute to severe immunopathology through excessive proinflammatory cytokine production, in addition to their role in leukocyte recruitment. Following H5N1 infection, AMs exhibited a reduced expression of the T-cell-recruiting chemokines CXCL9 and CXCL16 relative to H1N1 infection (Fig. S3B). Moreover, spatial cell communication analysis revealed a significant decrease in the number of interacting AMs and T cells mediated by the H2-D1-CD8B1 ligand-receptor pair, a component of the MHC class I signaling pathway, in the lungs of H5N1-infected mice compared to H1N1-infected controls (Fig. S5E). Collectively, these results suggest that H5N1 infection correlates with reduced adaptive immune activation, although the underlying mechanisms remain to be determined.

Furthermore, we investigated genes uniquely upregulated in either H5N1 or H1N1 infection (Fig. S4C). H5N1-specific DEGs were significantly enriched in biosynthetic and metabolic processes, whereas those exclusive to H1N1 were predominantly associated with immune defense pathways (Fig. S4D). This differential transcriptional profile may underlie the pronounced pulmonary damage characteristic of H5N1 infection. KEGG enrichment analysis of cell death-related DEGs revealed subset-specific patterns: Cluster 5 (metallothionein-expressing) was enriched in ferroptosis-related pathways; other clusters were associated with necroptosis and apoptosis; Cluster 0 showed no significant enrichment. Protein-protein interaction analysis indicated that all three cell death modalities are modulated by the P53 signaling pathway (Fig. S4E), suggesting that the influenza virus may promote AM depletion via P53-mediated mechanisms.

### Monocyte dysregulation in severe H5N1 infection

**Fig 7:**
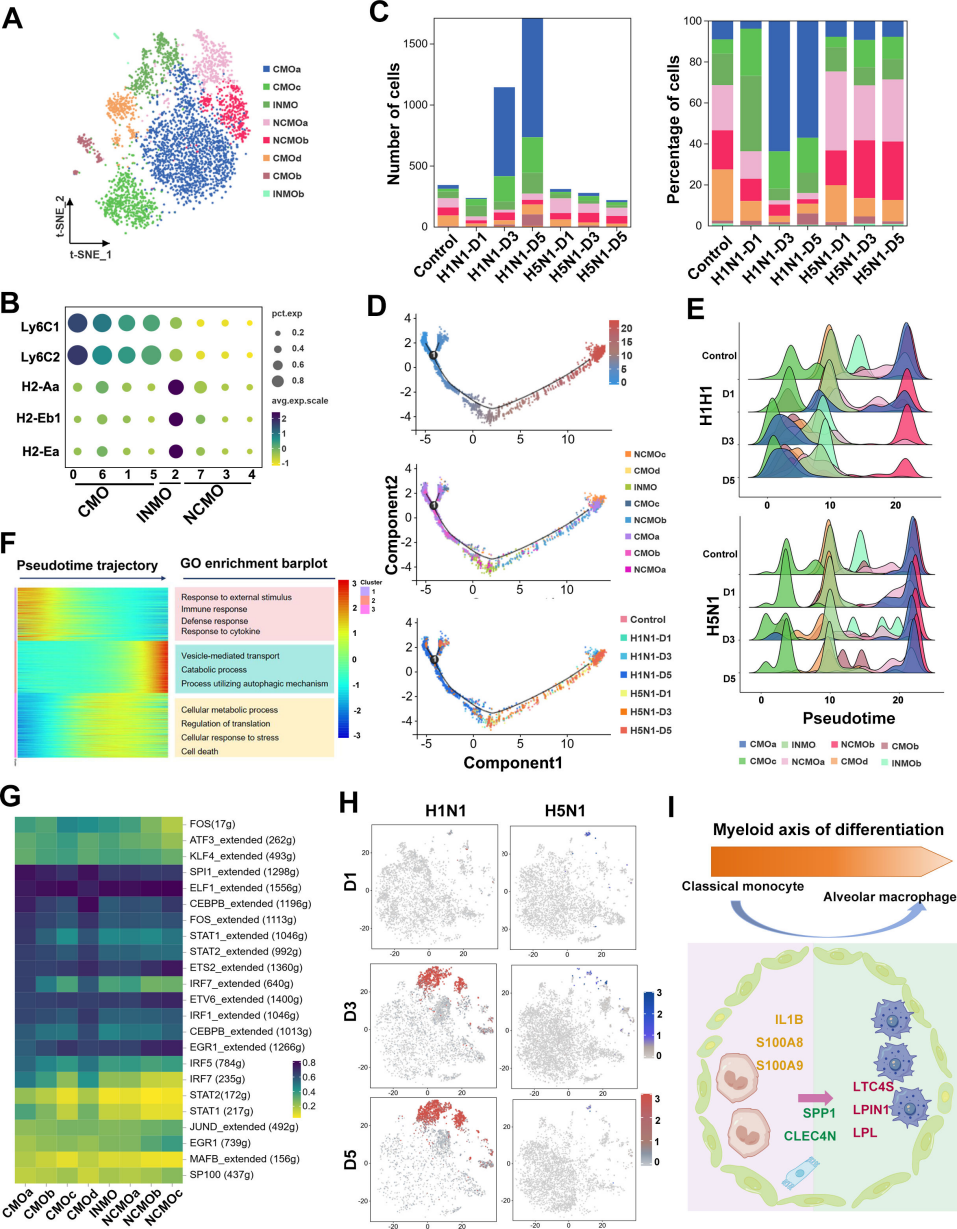
Immunological profiling of monocytes in the lungs of IAV-infected mice. (**A**) t-SNE visualization of reclustered monocytes from control, H1N1-, and H5N1-infected groups, revealing eight subclusters. (**B**) Dot plot showing expression of canonical markers for CMO, IMO, and NCMO clusters. (**C**) Absolute counts (left) and proportional abundance (right) of monocyte subsets per sample. (**D**) Pseudotime trajectory analysis of monocyte differentiation, colored by pseudotime (top), subcluster (middle), and sample origin (bottom). (**E**) Ridge plots showing the abundance dynamics of monocyte subsets along pseudotime for each sample group. (**F**) GO biological process terms enriched for genes differentially expressed at key trajectory branch points. (**G**) Heatmap depicting transcription factor regulon activity across monocyte subsets along pseudotime. (**H**) t-SNE plot highlighting the integration of classical monocytes and alveolar macrophages, showing infiltration of monocyte-derived macrophages. (**I**) Schematic diagram illustrating the shift from an inflammatory to a lipid metabolism signature during classical monocyte-to-macrophage differentiation.

Monocytes are recruited to tissue sites through chemokine-receptor interactions, where they secrete cytokines and chemokines that amplify inflammatory responses. To characterize monocyte dynamics during infection, we re-clustered monocytes into eight distinct subsets (Fig. 7A). Based on Ly6C and MHCII expression, these were categorized into three major populations: classical monocytes (CMOs; Ly6Cʰⁱ, Cluster 0/1/5/6), intermediate monocytes (IMOs; Ly6CⁱⁿᵗMHCIIʰⁱ, Cluster 2), and non-classical monocytes (NCMOs; Ly6Cˡᵒʷ, Cluster 3/4/7) (Fig. 7B). Cluster 1 was characterized by elevated expression of inflammatory genes (S100A8, S100A9, and IL1B), indicative of neutrophil activation (Fig. S6A). Following infection, S100 gene expression declined over time in H1N1-infected mice but remained significantly and persistently upregulated in H5N1 infection (Fig. S6B). This subset resembled the HLA-DRˡᵒʷS100Aʰⁱᵍʰ immunoregulatory monocytes described in severe COVID-19 (29). Clusters 0 and 6 showed elevated ISG expression, indicative of antiviral activity, while Cluster 5 demonstrated high CCR2 expression, suggesting a role in monocyte migration. Cluster 2 was marked by high expression of H2-Ab1, H2-Eb1, CD63, C1QA, C1QB, C1QC, and APOE, with further elevation of complement-related genes in Cluster 7—consistent with a transitional phenotype between monocytes, macrophages, and dendritic cells (4, 30). Clusters 3 and 4 exhibited upregulation of lipid metabolism-associated genes (LPL and LPIN1) and the nuclear receptor EAR2 (31, 32), reflecting a macrophage-like transcriptional signature reminiscent of monocyte subsets undergoing macrophage differentiation in severe COVID-19 (33). These data suggest that H5N1-infected monocytes may also exert immunosuppressive functions.

Comparative analysis revealed distinct monocyte profiles between H1N1 and H5N1 infections (Fig. 7C). H1N1 infection induced progressive expansion of CMOs, accompanied by enhanced antigen presentation shortly after viral exposure, followed by a temporal increase in interferon-responsive subsets. In contrast, H5N1 infection resulted in only a mild increase in inflammatory monocytes, indicating delayed activation. However, upregulation of macrophage differentiation markers such as EAR2 and LPL suggested alternative activation pathways (Fig. S6A). Together, these findings suggest that H5N1 infection may be associated with immunosuppressive functions in monocytes.

Pseudotime analysis reconstructed the differentiation trajectory of murine pulmonary monocytes, revealing progression from classical to intermediate and non-classical subsets. Infection with influenza strains of differing pathogenicity resulted in divergent differentiation biases: H1N1 infection favored an early differentiation phenotype, whereas H5N1 infection promoted features of late differentiation (Fig. 7D and E), aligning with observed heterogeneity in monocyte subclustering.

GO enrichment analysis of pseudotemporally ordered DEGs revealed that Cluster 1 genes (highly expressed early) were enriched in response to external stimuli and immune defense, while Clusters 2 and 3 genes (upregulated later) were involved in metabolic regulation, intracellular transport, and cell death (Fig. 7F). Regulon activity analysis further delineated temporal heterogeneity in cellular identity. Classical monocytes exhibited high activity of interferon-response transcription factors (IRF1, IRF5, and IRF7) (Fig. 7G). Along the differentiation axis, MAFB expression decreased while EGR1 increased. FOS expression initially rose, then declined, culminating in upregulation of its downstream target JUND. Upregulation of c-FOS and c-JUN during monocyte-to-macrophage differentiation enhances transcription of macrophage-associated genes, facilitating this cellular transition.

Recent studies have demonstrated the capacity of inflammatory monocytes (CMOs) to replenish AMs following acute lung injury (34). To evaluate infiltration of monocytes into the alveolar macrophage niche, we integrated CMO and AM subsets (Fig. 7H). Infection-induced tissue damage prompted continuous recruitment of inflammatory monocytes, which subsequently contributed to the generation of new macrophages. Notably, H1N1-infected lungs exhibited higher infiltration by monocyte-derived macrophages than H5N1-infected lungs, likely attributable to greater loss of tissue-resident AMs and enhanced monocyte recruitment following H1N1 infection. Pseudotime analysis delineated a differentiation trajectory from CMOs to AMs, indicating a phenotypic transition from an inflammatory signature toward metabolic reprogramming (Fig. 7I; Fig. S6C). Furthermore, transcriptomic analysis revealed that H1N1 infection resulted in upregulation of 1,551 genes in CMOs, whereas H5N1 infection led to upregulation of only 218 genes relative to controls (Fig. S6D). GO enrichment analysis indicated significant enrichment of immune regulation and defense responses to viral stimuli (Fig. S6E). Among DEGs associated with these terms, IAV infection induced upregulation of key immune genes (MX1, ISG15, IFITM3, IRF7, IFI44, ZBP1, CCL2, CCL5, IFNAR1, and IFNGR2), but expression levels were markedly lower in H5N1 than in H1N1 infection (Fig. S6F). These findings suggest that highly pathogenic H5N1 infection is associated with significantly reduced expression of interferons, cytokines, and chemokines compared to seasonal H1N1 infection, which may indicate attenuated immune activation in monocytes.

### Dysregulated neutrophil responses in severe H5N1 influenza infection

**Fig 8:**
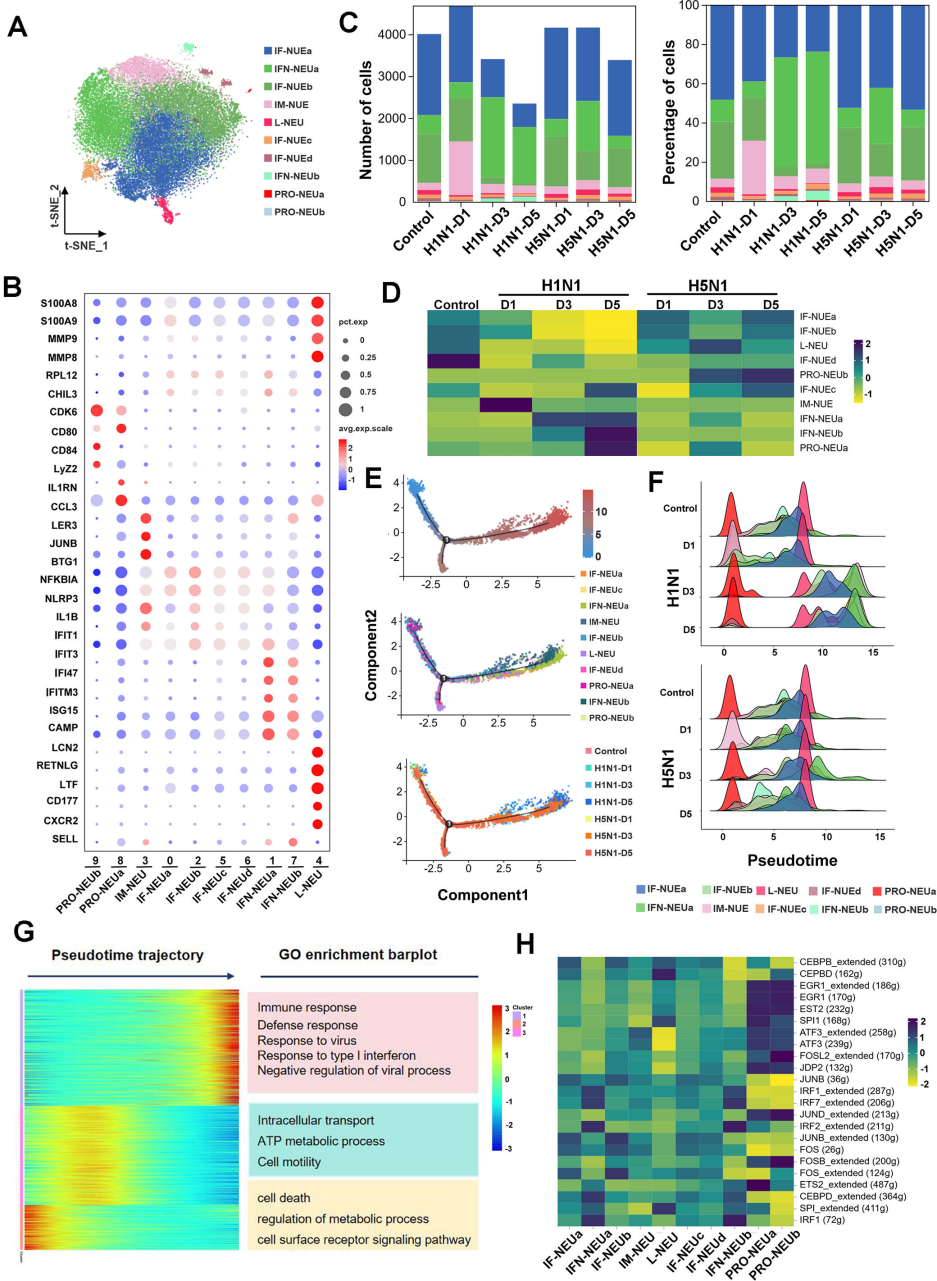
Immunological profiling of neutrophils in the lungs of IAV-infected mice. (**A**) t-SNE visualization of reclustered neutrophils from control, H1N1-, and H5N1-infected groups, revealing 10 subclusters. (**B**) Dot plot showing marker genes for IF-NEU, IFN-NEU, and L-NEU clusters. (**C**) Absolute counts (left) and proportional abundance (right) of neutrophil subsets per sample. (**D**) Heatmap showing the frequency of all neutrophil subclasses in each sample. (**E**) Pseudotime trajectory analysis of neutrophil differentiation, colored by pseudotime (top), subcluster (middle), and sample origin (bottom). (**F**) Ridge plots showing the abundance dynamics of neutrophil subsets along pseudotime for each sample group. (**G**) GO biological process terms enriched for genes differentially expressed at key trajectory branch points. (**H**) Heatmap depicting transcription factor regulon activity across neutrophil subclasses along pseudotime.

Neutrophils are the primary immune cells recruited from circulation to infection sites following viral exposure. Subclustering analysis identified substantial heterogeneity within the neutrophil compartment, defining ten transcriptionally distinct subtypes (Fig. 8A through C). Two precursor clusters (PRO-NEU, Clusters 8 and 9) exhibited elevated expression of CHIL3 and RPL12. An immature neutrophil cluster (IM-NEU, Cluster 3) was characterized by markers associated with neutrophil recruitment (CCL3) (35) and early inflammatory mediators, including IER3 (36). Two interferon-stimulated clusters (IFN-NEU, Clusters 1 and 7) displayed high ISG expression. Four inflammatory clusters (IF-NEU, Clusters 0, 2, 5, and 6) showed upregulated expression of IL1B, NLRP3, and NF-κB pathway genes. Cluster 4, which exhibited high lipocalin-2 (LCN2) expression, was classified as a low-density lipoprotein-related neutrophil subset (L-NEU). Despite its immature state, its CXCR2ˡᵒʷSELL^−^ (CD62L) phenotype (37) suggested an activated profile.

Comparative analysis revealed marked differences in neutrophil subset frequencies between H1N1 and H5N1 infections. Following H1N1 infection, mature neutrophils were enriched in interferon-associated clusters, indicative of a robust antiviral response. In contrast, H5N1 infection resulted in increased frequencies of inflammatory clusters IF-NEUa/b within the lung, despite overall lower cytokine levels, suggesting a sustained but attenuated inflammatory environment (Fig. 5D and 8D; Fig. S3C). Notably, Cluster 9 was exclusively detected in H5N1-infected samples. Neutrophils in this cluster exhibited upregulation of CD80, reduced CXCR2 expression (38), and elevated levels of the myeloid-derived suppressor cell (MDSC)-like marker CD84 (39). CXCR2 downregulation implies impaired chemotactic responsiveness. These dysregulated neutrophil phenotypes, marked by co-expression of activation and immunosuppressive markers, closely resemble those observed in severe sepsis (40). Previous studies indicate that such neutrophil subsets exhibit compromised antiviral activity, leading to inadequate viral clearance (38).

Pseudotime analysis revealed distinct differentiation trajectories for the L-NEU cluster and inflammatory interferon-responsive subsets. Neutrophils from H5N1-infected mice were positioned earlier along the pseudotime trajectory and exhibited a bias toward an immature, activated phenotype (Fig. 8E and F). Gene expression dynamics identified upregulation of genes involved in proliferation and differentiation (CXCR2 and MMP9) (Fig. S7A), supporting the prevalence of immature neutrophil states in H5N1 infection.

GO enrichment analysis of pseudotime-ordered DEGs from each cluster-specific gene set revealed distinct functional profiles (Fig. 8G). Genes in Cluster 1, highly expressed at the trajectory endpoint, were enriched in immune and defense responses. Cluster 2 genes, peaking early, were associated with metabolic processes, cellular communication, and cell death. Cluster 3 genes, maximally expressed mid-trajectory, participated in intracellular transport and energy metabolism.

Transcription factor activity dynamics during neutrophil differentiation showed that precursor clusters exhibited elevated expression of TFs associated with development and differentiation (EGR1, ETS2, SPI1, and ATF3) (Fig. 8H). ATF3, an early-response gene induced under stress, regulates neutrophil recruitment and aggregation (41). Immature and inflammatory clusters shared high expression of JDP2, JUND, and JUNB, while the maturation-associated gene FOS was upregulated in inflammatory mature clusters (42). Interferon-responsive regulons (IRF1, IRF2, and IRF7) were highly active in ISG-associated clusters. The L-NEU subset displayed a TF profile similar to precursor cells, consistent with an early differentiation state.

Among all immune subsets examined, neutrophils displayed the most pronounced inflammatory response to H5N1 infection. To investigate this, we focused our downstream analysis on interferon-stimulated neutrophil (IF-NEU) subpopulations. Comparative transcriptomics revealed 210 and 179 upregulated genes in H1N1- and H5N1-infected neutrophils, respectively, relative to controls (Fig. S7B). GO enrichment analysis highlighted stark functional differences: H1N1 infection drove the upregulation of immune-related processes, including viral regulation and cytokine signaling. Conversely, genes upregulated in H5N1 infection were primarily linked to metabolic processes and cell death, with minimal enrichment of immune pathways (Fig. S7C). These findings suggest that H5N1 infection may be associated with reduced host immune activation in neutrophils, which could potentially affect antiviral defense.

### The monocyte-macrophage axis in H1N1 and H5N1 influenza infection

**Fig 9:**
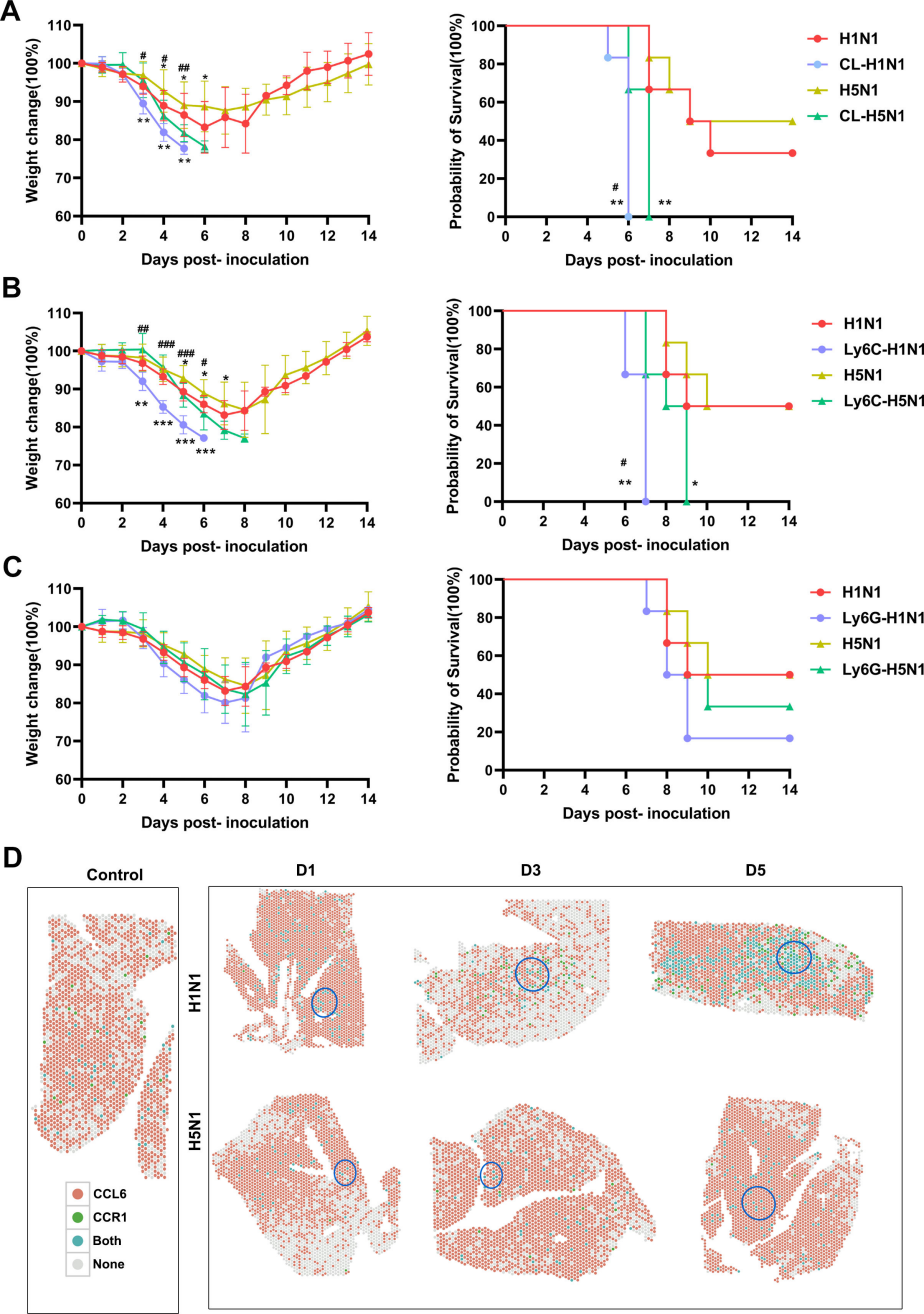
*In vivo* cell depletion. (**A**) BALB/c mice (*n* = 6) were treated with clodronate liposomes or PBS liposomes at 48 h before and 24 h after viral challenge to deplete alveolar macrophages, then challenged with 1× MLD_50_ of H1N1 or H5N1. Body weight change (left) and survival (right) were monitored daily for 14 days. (**B and C**) Mice (*n* = 6) were treated with anti-Ly6C (**B**), anti-Ly6G (**C**), or isotype control antibodies at 24 h before and 48 h after viral challenge to deplete monocytes or neutrophils, respectively, then challenged with 1× MLD_50_ of H1N1 or H5N1. Body weight change (left) and survival (right) were monitored daily. Significance is indicated as *Control vs. IAV; #H1N1 vs. H5N1. (**D**) Spatial visualization of CCL6-CCR1 ligand-receptor-mediated communication between AMs and monocytes in control, H1N1-, and H5N1-infected groups, as mapped by CellTrek.

During lung injury, resident AMs release pro-inflammatory cytokines that recruit circulating neutrophils and monocytes, perpetuating inflammation and tissue damage. To delineate the functional roles of specific myeloid subsets in influenza pathogenesis, we selectively depleted alveolar macrophages using clodronate liposomes (administered 48 h before and 24 h after viral infection), and depleted neutrophils or monocytes using anti-Ly6G or anti-Ly6C-neutralizing antibodies (administered 24 h before and 48 h after viral infection) (Fig. S8A). Depletion efficiency, validated by flow cytometry, exceeded 85% for all three populations (Fig. S8B). Subsequent viral challenge revealed that monocyte and macrophage depletion led to accelerated weight loss and increased mortality. In contrast, neutrophil depletion had no significant effect on disease outcome (Fig. 9A through C). Notably, H1N1-infected mice exhibited more rapid disease progression, consistent with scRNA-seq data showing substantial AM loss and monocyte recruitment. These results underscore the critical role of the monocyte-macrophage axis in influenza pathogenesis.

Given this, we hypothesized that inhibitory monocytes might regulate disease progression by modulating T-cell responses. To test this, we first reclustered T cells and annotated subsets based on canonical markers (Fig. S9A). This identified naïve and central memory CD4+ T cells, Tregs, Th2, Th17, and CCL4-expressing CD4+ T cells, as well as cytotoxic, naïve, exhausted, and effector memory CD8+ T cells (Fig. S9A and B). We then examined communication between the putative inhibitory monocyte subsets (CMOc and NCMOb/c) and these T-cell populations. Following H5N1 infection, both the number and strength of signaling interactions with T cells were markedly lower than in H1N1 infection, particularly with Th2, Th17, CD4 Tcm, and cytotoxic CD8 T cells (Fig. S9C). This suggests that impaired adaptive immune activation, potentially driven by dysfunctional T-cell responses, may contribute to the immunosuppressive phenotype observed in H5N1 infection.

To further elucidate spatial immune dynamics, we mapped the CCL6-CCR1 ligand-receptor pair, identified in our scRNA-seq data, onto spatial transcriptomic profiles. This revealed a temporal increase in AM-monocyte interactions in H1N1-infected lungs, a phenomenon absent in H5N1-infected tissue (Fig. 9D). This spatial analysis reinforces the concept of impaired innate immune activation during H5N1 infection. Collectively, these findings highlight distinct, spatially organized immune modulation mechanisms centered on the monocyte-macrophage axis that differentiate the host response to seasonal and highly pathogenic influenza viruses.

## DISCUSSION

Influenza virus remains a persistent threat to global public health, contributing significantly to morbidity and mortality. A detailed understanding of the host immune response is essential for developing effective therapeutic strategies. However, key aspects of the immune response to IAV in the lung, particularly the roles of innate immune populations and the mediators of immunopathogenesis, remain incompletely characterized. The integration of scRNA-seq and spatial transcriptomics offers a powerful approach to systematically investigate the spatiotemporal dynamics of host interactions with both seasonal and HPAI viruses.

Our initial findings revealed that despite the significantly lower MLD_50_ of H5N1 in mice, it did not demonstrate superior pathogenic potential compared to H1N1 when administered at equivalent lethal doses, instead exhibiting delayed replication kinetics. We hypothesized that this disparity is linked to viral receptor-binding preferences. The avian-origin H5N1 strain primarily targets α2,3-linked sialic acid receptors in the lower respiratory tract (1), whereas the seasonal H1N1 virus preferentially binds α2,6-linked receptors in the upper airways. Notably, the 2009 pandemic H1N1 strain used here also retains affinity for α2,3-linked receptors (43), which may facilitate the maintenance of high viral titers in the lung. These distinct entry portals may contribute to divergent infection strategies: HPAI viruses rapidly breach innate barriers to achieve systemic dissemination before adaptive immunity is fully mobilized, often with fatal consequences. Seasonal viruses, in contrast, have evolved for more efficient early infection and homeostatic replication. These fundamental differences are likely to elicit divergent host immune responses, ultimately shaping disparate disease progression and clinical outcomes.

Building on this, we integrated scRNA-seq and spatial transcriptomics to construct a high-resolution dynamic atlas of the pulmonary immune microenvironment during H1N1 and H5N1 infection. Analysis of 84,162 cells identified 24 distinct clusters representing 12 immune cell types, and spatial transcriptomics enabled the spatial mapping of these populations within the native tissue architecture. This integrated approach revealed starkly contrasting immune response patterns. H1N1 infection was associated with robust, multi-lineage interferon responses. Neutrophils and monocytes exhibited pronounced inflammatory activation, with extensive chemokine-mediated crosstalk, particularly involving AMs that expressed diverse chemokine receptors.

Despite their shared pro-inflammatory roles, H1N1 and H5N1 induced divergent cytokine profiles. Counter to the paradigm that H5N1 triggers a dysregulated cytokine storm leading to ARDS (44), we observed an attenuated pulmonary inflammation and reduced interferon responses. While H5N1-infected AMs did show elevated levels of the pro-inflammatory cytokines IL-1α and IL-1β, this was associated with an overall reduction in the broader immune response. This finding aligns with studies of severe COVID-19, which also report lower cytokine levels and suppressed interferon signaling compared to severe influenza (45). Notably, cytokine storms can be triggered by low-pathogenicity avian influenza viruses (H9N2 [46], H10N3 [47], H10N8 [48]), and most severe COVID-19 patients do not exhibit cytokine storm phenotypes, suggesting this mechanism may not be the primary cause of H5N1 pathogenicity. Nevertheless, we identified a potentially pathogenic role for a metabolically distinct AM subset (M-AMs) expressing leukotriene C4 synthase (LTC4S). This subset progressively declined during H1N1 infection but persisted during H5N1 infection. Conversely, H1N1 infection promoted the expansion of interferon-responsive AMs with antiviral functions—a response notably absent in H5N1. This differential recruitment of protective versus pathogenic macrophages may explain the distinct cytokine profiles. Furthermore, H5N1 infection was associated with reduced expression of T cell-recruiting chemokines CXCL9 and CXCL16 by AMs and fewer H2-D1-CD8B1 ligand-receptor interactions *in situ*, suggesting less efficient adaptive immune activation. The enrichment of a metallothionein-rich AM cluster (MT-AM) in H5N1-infected mice, also observed in severe COVID-19 (25) and advanced COPD (49), points to shared immune dysregulation patterns across severe pulmonary diseases.

Monocyte subsets also exhibited severity- and time-dependent alterations. H5N1 infection was marked by an expansion of non-classical monocytes involved in late-stage differentiation and metabolic regulation. In contrast, H1N1 infection was characterized by CMOs displaying robust inflammatory and interferon responses, which differentiated into Mo-AMs—a population known to be associated with more severe cytokine storms than tissue-resident AMs. A specific classical monocyte subset (CMOd), enriched in H5N1 infection, expressed high levels of PLAC8, a gene associated with poor prognosis in sepsis (50) that suppresses IL-1β and IL-18 (51). This subset also expressed high levels of NR4A1, which can promote macrophage differentiation with enhanced antigen presentation capabilities (52). The progressive decline of this potentially dysfunctional CMOd subset during H1N1 infection may contribute to its more effective antiviral response. In H5N1 infection, we observed monocytes with an immunoregulatory (S100A8/9, MMP9) (29) and alternative activation (EAR2, LPL) signature (33), alongside a global downregulation of genes associated with cytokine and interferon production. This profile suggests a state of reduced immune responsiveness, characterized by a loss of monocyte-derived dendritic cells and the emergence of MDSC-like monocytes. Critically, communication between these MDSC-like monocytes and CD8^+^ cytotoxic T lymphocytes was reduced in H5N1-infected mice. As CD8^+^ T cells are essential for viral clearance, this disruption may explain the progressive increase in viral titers observed during H5N1 infection. Although functional validation was not performed in the current study, we have acknowledged this limitation and propose that future experiments using adoptive transfer or genetic ablation models (focusing on subsets such as Treg, Th1/2, and CD8 + T cells) will be critical to functionally validate their immunosuppressive roles.

A similar pattern of dysregulation was evident in the neutrophil compartment. An L-NEU subset, exhibiting an immature, activated phenotype and elevated LCN2, expanded during H5N1 infection but progressively declined during H1N1 infection. Persistent LCN2 expression, a hallmark of innate immune dysfunction and emergency granulopoiesis in sepsis (53), and the expansion of low-density neutrophils correlate with COVID-19 severity (33, 54). H5N1-infected hosts also showed enrichment of neutrophil precursors expressing MDSC markers like CD84 (39) and displaying reduced CXCR2 expression, a feature associated with impaired migration in severe sepsis and COVID-19 (54, 55). These subsets, generated via emergency granulopoiesis, are closely linked to altered immune function. Conversely, an interferon-responsive neutrophil population (IFN-NEU) with high ISG15 and IFITM3 expression, implicated in antiviral defense (56), was significantly expanded during H1N1 infection but showed only a modest, transient increase during H5N1 infection. Collectively, the dysregulated neutrophil response in H5N1 infection, characterized by emergency myelopoiesis and an altered inflammatory phenotype, likely contributes to the severity of lung injury.

*In vivo* depletion experiments underscored the functional importance of these myeloid populations. Removal of AMs and monocytes exacerbated mortality in both infections, with a more pronounced effect in H1N1-infected mice, likely due to its greater cellular heterogeneity and more extensive intercellular communication. The transcription factor NR4A1 was enriched in both an inflammatory AM subset and CCR2^+^ monocytes. Given its known role as a key regulator of T-cell dysfunction (57) and its association with MDSC phenotypes, NR4A1 presents a compelling therapeutic target. Notably, a PROTAC targeting NR4A1 (NR-V04) can reverse MDSC phenotypes in monocytes and promote effector memory T-cell responses (58), while the NR4A1 agonist cytosporone B can control influenza virus infection and improve lung function (59, 60).

In summary, this study delineates the spatiotemporal dynamics of immune responses to seasonal and highly pathogenic avian influenza viruses. Seasonal H1N1 infection elicits a robust, multi-faceted inflammatory and interferon response associated with specific subsets of AMs, monocytes, and neutrophils. In contrast, HPAIV H5N1 infection is characterized by a restrained cytokine response, profoundly reduced interferon signaling, defective monocyte activation, dysregulated myelopoiesis, and reduced T-cell activation. These findings underscore the critical role of the monocyte-macrophage axis in IAV immunopathogenesis and highlight NR4A1 as a potential therapeutic target for severe influenza virus pneumonia.

### Study limitations

We acknowledge several limitations in this study. First, all subsequent experiments were conducted using a single fixed viral dose. This approach enabled in-depth interrogation under conditions of equivalent lethality but did not capture dose-dependent variations in host responses. Moreover, dose equivalence between H1N1 and H5N1 is an approximation, as differences in virus stock properties could affect the interpretation of dose-response relationships. Second, our internalization assay did not include a blocking control to definitively confirm that viral uptake occurs via phagocytosis, nor did we characterize defective interfering particles (DIPs) in our viral stocks, which have been shown to influence replication and immune responses in a context-dependent manner (61). Formal demonstration of these mechanisms will require additional experiments. Third, the spatial transcriptomics analysis was performed without biological replicates. Our primary objective was to map the anatomical localization of immune subsets, not to perform statistical comparisons. Fourth, our findings on cytokine responses, intercellular communication, and pseudotime trajectories are primarily derived from transcriptomic data. Protein-level validation and functional validation of predicted interactions were not performed and will be essential for establishing causality. Fifth, we attempted to map influenza viral reads in our scRNA-seq data but detected extremely low levels, consistent with the fact that immune cells are not primary targets of influenza virus replication. Therefore, we could not definitively determine which immune cell populations were directly infected. Sixth, our findings are derived from a single H5N1 viral strain (clade 2.3.2.1) in a mouse model and may not fully reflect the heterogeneity of H5N1 clades or the complexity of human disease. Future studies incorporating multiple viral isolates, graded doses, and larger sample sizes are necessary to determine the broader applicability of our conclusions.

## Data Availability

The raw single‑cell RNA sequencing data generated in this study have been deposited in the Genome Sequence Archive (GSA) at the National Genomics Data Center (CNCB/NGDC) under BioProject accession number PRJCA063779 and GSA accession number CRA042735. The complete genome sequence of the H5N1 virus strain (clade 2.3.2.1) has been deposited in GenBank under accession numbers PZ374593 (HA), PZ374594 (NA), PZ484986 (PB2), PZ484987 (PB1), PZ484988 (PA), PZ484989 (NP), PZ484990 (NS), and PZ484991 (M). All other data supporting the findings of this study are available from the corresponding author upon reasonable request. All custom code is available upon request.

## SUPPLEMENTAL MATERIAL

Supplemental figures jvi.00746-26-s0001.pdf Fig. S1 to S9.

Supplemental tables jvi.00746-26-s0002.docx Tables S1 to S7.

ASM does not own the copyrights to Supplemental Material that may be linked to, or accessed through, an article. The authors have granted ASM a non-exclusive, world-wide license to publish the Supplemental Material files. Please contact the corresponding author directly for reuse.

## References

[1] Xing X, Shi J, Cui P, Yan C, Zhang Y, Zhang Y, Wang C, Chen Y, Zeng X, Tian G, Liu L, Guan Y, Li C, Suzuki Y, Deng G, Chen H. 2024. Evolution and biological characterization of H5N1 influenza viruses bearing the clade 2.3.2.1 hemagglutinin gene. Emerg Microbes Infect 13:2284294. doi:10.1080/22221751.2023.2284294 37966008 PMC10769554

[2] van Riel D, Leijten LME, van der Eerden M, Hoogsteden HC, Boven LA, Lambrecht BN, Osterhaus ADME, Kuiken T. 2011. Highly pathogenic avian influenza virus H5N1 infects alveolar macrophages without virus production or excessive TNF-alpha induction. PLoS Pathog 7:e1002099. doi:10.1371/journal.ppat.1002099 21731493 PMC3121882

[3] David C, Verney C, Si-Tahar M, Guillon A. 2024. The deadly dance of alveolar macrophages and influenza virus. Eur Respir Rev 33:240132. doi:10.1183/16000617.0132-2024 39477353 PMC11522969

[4] Aegerter H, Kulikauskaite J, Crotta S, Patel H, Kelly G, Hessel EM, Mack M, Beinke S, Wack A. 2020. Influenza-induced monocyte-derived alveolar macrophages confer prolonged antibacterial protection. Nat Immunol 21:145–157. doi:10.1038/s41590-019-0568-x 31932810 PMC6983324

[5] Kulasinghe A, Tan CW, Ribeiro Dos Santos Miggiolaro AF, Monkman J, SadeghiRad H, Bhuva DD, Motta Junior J da S, Busatta Vaz de Paula C, Nagashima S, Baena CP, Souza-Fonseca-Guimaraes P, de Noronha L, McCulloch T, Rossi GR, Cooper C, Tang B, Short KR, Davis MJ, Souza-Fonseca-Guimaraes F, Belz GT, O’Byrne K. 2022. Profiling of lung SARS-CoV-2 and influenza virus infection dissects virus-specific host responses and gene signatures. Eur Respir J 59:2101881. doi:10.1183/13993003.01881-2021 34675048 PMC8542865

[6] Zhang Y, Xing X, Long B, Cao Y, Hu S, Li X, Yu Y, Tian D, Sui B, Luo Z, Liu W, Lv L, Wu Q, Dai J, Zhou M, Han H, Fu ZF, Gong H, Bai F, Zhao L. 2022. A spatial and cellular distribution of rabies virus infection in the mouse brain revealed by fMOST and single‐cell RNA sequencing. Clinical & Translational Med 12:e700. doi:10.1002/ctm2.700 PMC8776042 35051311

[7] He M-Y, Liu M, Yuan J, Lv J, Li W, Yan Q, Tang Y, Wang L, Guo L, Liu F. 2025. Spatial transcriptomics reveals tumor microenvironment heterogeneity in EBV positive diffuse large B cell lymphoma. Sci Rep 15:15878. doi:10.1038/s41598-025-00410-x 40335578 PMC12058988

[8] Kudo E, Song E, Yockey LJ, Rakib T, Wong PW, Homer RJ, Iwasaki A. 2019. Low ambient humidity impairs barrier function and innate resistance against influenza infection. Proc Natl Acad Sci USA 116:10905–10910. doi:10.1073/pnas.1902840116 31085641 PMC6561219

[9] Steuerman Y, Cohen M, Peshes-Yaloz N, Valadarsky L, Cohn O, David E, Frishberg A, Mayo L, Bacharach E, Amit I, Gat-Viks I. 2018. Dissection of influenza infection in vivo by single-Cell RNA sequencing. Cell Syst 6:679–691. doi:10.1016/j.cels.2018.05.008 29886109 PMC7185763

[10] Jiang Z, Wei F, Zhang Y, Wang T, Gao W, Yu S, Sun H, Pu J, Sun Y, Wang M, Tong Q, Gao C, Chang K-C, Liu J. 2021. IFI16 directly senses viral RNA and enhances RIG-I transcription and activation to restrict influenza virus infection. Nat Microbiol 6:932–945. doi:10.1038/s41564-021-00907-x 33986530

[11] Wei F, Jiang Z, Sun H, Pu J, Sun Y, Wang M, Tong Q, Bi Y, Ma X, Gao GF, Liu J. 2019. Induction of PGRN by influenza virus inhibits the antiviral immune responses through downregulation of type I interferons signaling. PLoS Pathog 15:e1008062. doi:10.1371/journal.ppat.1008062 31585000 PMC6795447

[12] Liu Z, Gu Y, Shin A, Zhang S, Ginhoux F. 2020. Analysis of myeloid cells in mouse tissues with flow cytometry. STAR Protoc 1:100029. doi:10.1016/j.xpro.2020.100029 33111080 PMC7580097

[13] Wilk AJ, Rustagi A, Zhao NQ, Roque J, Martínez-Colón GJ, McKechnie JL, Ivison GT, Ranganath T, Vergara R, Hollis T, Simpson LJ, Grant P, Subramanian A, Rogers AJ, Blish CA. 2020. A single-cell atlas of the peripheral immune response in patients with severe COVID-19. Nat Med 26:1070–1076. doi:10.1038/s41591-020-0944-y 32514174 PMC7382903

[14] Saleh KK, Xi H, Switzler C, Skuratovsky E, Romero MA, Chien P, Gibbs D, Gane L, Hicks MR, Spencer MJ, Pyle AD. 2022. Single cell sequencing maps skeletal muscle cellular diversity as disease severity increases in dystrophic mouse models. iScience 25:105415. doi:10.1016/j.isci.2022.105415 36388984 PMC9646951

[15] McGinnis CS, Murrow LM, Gartner ZJ. 2019. DoubletFinder: doublet detection in single-cell RNA sequencing data using artificial nearest neighbors. Cell Syst 8:329–337. doi:10.1016/j.cels.2019.03.003 30954475 PMC6853612

[16] Wei R, He S, Bai S, Sei E, Hu M, Thompson A, Chen K, Krishnamurthy S, Navin NE. 2022. Spatial charting of single-cell transcriptomes in tissues. Nat Biotechnol 40:1190–1199. doi:10.1038/s41587-022-01233-1 35314812 PMC9673606

[17] Jin S, Plikus MV, Nie Q. 2025. CellChat for systematic analysis of cell-cell communication from single-cell transcriptomics. Nat Protoc 20:180–219. doi:10.1038/s41596-024-01045-4 39289562

[18] Qiu X, Mao Q, Tang Y, Wang L, Chawla R, Pliner HA, Trapnell C. 2017. Reversed graph embedding resolves complex single-cell trajectories. Nat Methods 14:979–982. doi:10.1038/nmeth.4402 28825705 PMC5764547

[19] Tate MD, Brooks AG, Reading PC, Mintern JD. 2012. Neutrophils sustain effective CD8(+) T-cell responses in the respiratory tract following influenza infection. Immunol Cell Biol 90:197–205. doi:10.1038/icb.2011.26 21483446

[20] de Veer MJ, Holko M, Frevel M, Walker E, Der S, Paranjape JM, Silverman RH, Williams BRG. 2001. Functional classification of interferon-stimulated genes identified using microarrays. J Leukoc Biol 69:912–920. doi:10.1189/jlb.69.6.912 11404376

[21] Xiao K, Cao Y, Han Z, Zhang Y, Luu LDW, Chen L, Yan P, Chen W, Wang J, Liang Y, Shi X, Wang X, Wang F, Hu Y, Wen Z, Chen Y, Yang Y, Yu H, Xie L, Wang Y. 2025. A pan-immune panorama of bacterial pneumonia revealed by a large-scale single-cell transcriptome atlas. Signal Transduct Target Ther 10:5. doi:10.1038/s41392-024-02093-8 39757231 PMC11701081

[22] Moshkelgosha S, Duong A, Wilson G, Andrews T, Berra G, Renaud-Picard B, Liu M, Keshavjee S, MacParland S, Yeung J, Martinu T, Juvet S. 2022. Interferon-stimulated and metallothionein-expressing macrophages are associated with acute and chronic allograft dysfunction after lung transplantation. J Heart Lung Transplant 41:1556–1569. doi:10.1016/j.healun.2022.05.005 35691795

[23] Fuchs T, Puellmann K, Emmert A, Fleig J, Oniga S, Laird R, Heida NM, Schäfer K, Neumaier M, Beham AW, Kaminski WE. 2015. The macrophage-TCRαβ is a cholesterol-responsive combinatorial immune receptor and implicated in atherosclerosis. Biochem Biophys Res Commun 456:59–65. doi:10.1016/j.bbrc.2014.11.034 25446098

[24] Chávez-Galán L, Olleros ML, Vesin D, Garcia I. 2015. Much more than M1 and M2 macrophages, there are also CD169(+) and TCR(+) macrophages. Front Immunol 6:263. doi:10.3389/fimmu.2015.00263 26074923 PMC4443739

[25] Lee JTH, Barnett SN, Roberts K, Ashwin H, Milross L, Cho J-W, Huseynov A, Woodhams B, Aivazidis A, Li T, et al.. 2025. Integrated histopathology, spatial and single cell transcriptomics resolve cellular drivers of early and late alveolar damage in COVID-19. Nat Commun 16:1979. doi:10.1038/s41467-025-56473-x 40064844 PMC11893906

[26] Capucetti A, Albano F, Bonecchi R. 2020. Multiple roles for chemokines in neutrophil biology. Front Immunol 11:1259. doi:10.3389/fimmu.2020.01259 32733442 PMC7363767

[27] Lv C, Li Y, Wang T, Zhang Q, Qi J, Sima M, Li E, Qin T, Shi Z, Li F, Wang X, Sun W, Feng N, Yang S, Xia X, Jin N, Zhou Y, Gao Y. 2023. Taurolidine improved protection against highly pathogenetic avian influenza H5N1 virus lethal-infection in mouse model by regulating the NF-κB signaling pathway. Virol Sin 38:119–127. doi:10.1016/j.virs.2022.11.010 36450323 PMC10006309

[28] Liao Y, Fu Z, Huang Y, Wu S, Wang Z, Ye S, Zeng W, Zeng G, Li D, Yang Y, Pei K, Yang J, Hu Z, Liang X, Hu J, Liu M, Jin J, Cai C. 2023. Interleukin-18-primed human umbilical cord-mesenchymal stem cells achieve superior therapeutic efficacy for severe viral pneumonia via enhancing T-cell immunosuppression. Cell Death Dis 14:66. doi:10.1038/s41419-023-05597-3 36707501 PMC9883134

[29] Yao R-Q, Zhao P-Y, Li Z-X, Liu Y-Y, Zheng L-Y, Duan Y, Wang L, Yang R-L, Kang H-J, Hao J-W, Li J-Y, Dong N, Wu Y, Du X-H, Zhu F, Ren C, Wu G-S, Xia Z-F, Yao Y-M. 2023. Single-cell transcriptome profiling of sepsis identifies HLA-DRlowS100Ahigh monocytes with immunosuppressive function. Military Med Res 10:27. doi:10.1186/s40779-023-00462-y PMC10278311 37337301

[30] Cheong C, Matos I, Choi J-H, Dandamudi DB, Shrestha E, Longhi MP, Jeffrey KL, Anthony RM, Kluger C, Nchinda G, Koh H, Rodriguez A, Idoyaga J, Pack M, Velinzon K, Park CG, Steinman RM. 2010. Microbial stimulation fully differentiates monocytes to DC-SIGN/CD209(+) dendritic cells for immune T cell areas. Cell 143:416–429. doi:10.1016/j.cell.2010.09.039 21029863 PMC3150728

[31] Chen J, Huang XR, Yang F, Yiu WH, Yu X, Tang SCW, Lan HY. 2022. Single‐cell RNA sequencing identified novel Nr4a1 ^+^ Ear2 ^+^ anti‐inflammatory macrophage phenotype under myeloid‐TLR4 dependent regulation in anti‐glomerular basement membrane (GBM) Crescentic glomerulonephritis (cGN). Advanced Science 9. doi:10.1002/advs.202200668 PMC9218767 35484716

[32] Kumar S, Mickael C, Kumar R, Prasad RR, Campbell NV, Zhang H, Li M, McKeon BA, Allen TE, Graham BB, Yu Y-RA, Stenmark KR. 2024. Single cell transcriptomic analyses reveal diverse and dynamic changes of distinct populations of lung interstitial macrophages in hypoxia-induced pulmonary hypertension. Front Immunol 15:1372959. doi:10.3389/fimmu.2024.1372959 38690277 PMC11059952

[33] Schulte-Schrepping J, Reusch N, Paclik D, Baßler K, Schlickeiser S, Zhang B, Krämer B, Krammer T, Brumhard S, Bonaguro L, et al.. 2020. Severe COVID-19 is marked by a dysregulated myeloid cell compartment. Cell 182:1419–1440. doi:10.1016/j.cell.2020.08.001 32810438 PMC7405822

[34] Niethamer TK, Planer JD, Morley MP, Babu A, Zhao G, Basil MC, Cantu E, Frank DB, Diamond JM, Nottingham AN, Li S, Sharma A, Hallquist H, Levin LI, Zhou S, Vaughan AE, Morrisey EE. 2025. Longitudinal single-cell profiles of lung regeneration after viral infection reveal persistent injury-associated cell states. Cell Stem Cell 32:302–321. doi:10.1016/j.stem.2024.12.002 39818203 PMC11805657

[35] Bourdon JA, Halappanavar S, Saber AT, Jacobsen NR, Williams A, Wallin H, Vogel U, Yauk CL. 2012. Hepatic and pulmonary toxicogenomic profiles in mice intratracheally instilled with carbon black nanoparticles reveal pulmonary inflammation, acute phase response, and alterations in lipid homeostasis. Toxicol Sci 127:474–484. doi:10.1093/toxsci/kfs119 22461453 PMC3355316

[36] Küppers O, Ahmad M, Haffner-Luntzer M, Scharffetter-Kochanek K, Ignatius A, Fischer V. 2024. Inflammatory priming of human mesenchymal stem cells induces osteogenic differentiation via the early response gene IER3. FASEB J 38:e70076. doi:10.1096/fj.202401344R 39373973

[37] Caxaria S, Bharde S, Fuller AM, Evans R, Thomas B, Celik P, Dell’Accio F, Yona S, Gilroy D, Voisin M-B, Wood JN, Sikandar S. 2023. Neutrophils infiltrate sensory ganglia and mediate chronic widespread pain in fibromyalgia. Proc Natl Acad Sci USA 120:e2211631120. doi:10.1073/pnas.2211631120 37071676 PMC10151464

[38] Gong HH, Worley MJ, Carver KA, Godin CJ, Deng JC. 2025. Deficient neutrophil responses early in influenza infection promote viral replication and pulmonary inflammation. PLoS Pathog 21:e1012449. doi:10.1371/journal.ppat.1012449 39823516 PMC11845034

[39] Zhu Y, Murtadha M, Liu M, Caserta E, Napolitano O, Nguyen LXT, Wang H, Moloudizargari M, Nigam L, Tandoh T, et al.. 2025. Identification of CD84 as a potent survival factor in acute myeloid leukemia. J Clin Invest 135:e176818. doi:10.1172/JCI176818 40198133 PMC12126229

[40] van der Poll T, Shankar-Hari M, Wiersinga WJ. 2021. The immunology of sepsis. Immunity 54:2450–2464. doi:10.1016/j.immuni.2021.10.012 34758337

[41] Subramanian P, Mitroulis I, Hajishengallis G, Chavakis T. 2016. Regulation of tissue infiltration by neutrophils: role of integrin α3β1 and other factors. Curr Opin Hematol 23:36–43. doi:10.1097/MOH.0000000000000198 26554893 PMC4668230

[42] Wu F, Fan J, He Y, Xiong A, Yu J, Li Y, Zhang Y, Zhao W, Zhou F, Li W, Zhang J, Zhang X, Qiao M, Gao G, Chen S, Chen X, Li X, Hou L, Wu C, Su C, Ren S, Odenthal M, Buettner R, Fang N, Zhou C. 2021. Single-cell profiling of tumor heterogeneity and the microenvironment in advanced non-small cell lung cancer. Nat Commun 12:2540. doi:10.1038/s41467-021-22801-0 33953163 PMC8100173

[43] Bradley KC, Jones CA, Tompkins SM, Tripp RA, Russell RJ, Gramer MR, Heimburg-Molinaro J, Smith DF, Cummings RD, Steinhauer DA. 2011. Comparison of the receptor binding properties of contemporary swine isolates and early human pandemic H1N1 isolates (Novel 2009 H1N1). Virology 413:169–182. doi:10.1016/j.virol.2011.01.027 21353280

[44] de Jong MD, Simmons CP, Thanh TT, Hien VM, Smith GJD, Chau TNB, Hoang DM, Chau NVV, Khanh TH, Dong VC, Qui PT, Cam BV, Ha DQ, Guan Y, Peiris JSM, Chinh NT, Hien TT, Farrar J. 2006. Fatal outcome of human influenza A (H5N1) is associated with high viral load and hypercytokinemia. Nat Med 12:1203–1207. doi:10.1038/nm1477 16964257 PMC4333202

[45] Mudd PA, Crawford JC, Turner JS, Souquette A, Reynolds D, Bender D, Bosanquet JP, Anand NJ, Striker DA, Martin RS, Boon ACM, House SL, Remy KE, Hotchkiss RS, Presti RM, O’Halloran JA, Powderly WG, Thomas PG, Ellebedy AH. 2020. Distinct inflammatory profiles distinguish COVID-19 from influenza with limited contributions from cytokine storm. Sci Adv 6:eabe3024. doi:10.1126/sciadv.abe3024 33187979 PMC7725462

[46] Li Y, Quan X, Chen R, Wang X, Chen Y, Gan Y, Irwin DM, Shen Y. 2025. Adaptive selection of quasispecies during in vivo passaging in chickens, mice, and ferrets results in host-specific strains for the H9N2 avian influenza virus. J Virol 99:e0015125. doi:10.1128/jvi.00151-25 40338080 PMC12172485

[47] Wang X, Wang X, Hao X, Gao R, Lu X, Yang W, Chen Y, Hu J, Gu M, Liu X, Hu S, Liu K, Wang X, Liu X. 2025. The novel H10N3 avian influenza virus triggers lethal cytokine storm by activating multiple forms of programmed cell death in mammalian lungs. IJMS 26:1977. doi:10.3390/ijms26051977 40076601 PMC11899735

[48] Liu M, Li X, Yuan H, Zhou J, Wu J, Bo H, Xia W, Xiong Y, Yang L, Gao R, et al.. 2015. Genetic diversity of avian influenza A (H10N8) virus in live poultry markets and its association with human infections in China. Sci Rep 5:7632. doi:10.1038/srep07632 25591167 PMC5379002

[49] Sauler M, McDonough JE, Adams TS, Kothapalli N, Barnthaler T, Werder RB, Schupp JC, Nouws J, Robertson MJ, Coarfa C, et al.. 2022. Characterization of the COPD alveolar niche using single-cell RNA sequencing. Nat Commun 13:494. doi:10.1038/s41467-022-28062-9 35078977 PMC8789871

[50] Maslove DM, Shapira T, Tyryshkin K, Veldhoen RA, Marshall JC, Muscedere J. 2019. Validation of diagnostic gene sets to identify critically ill patients with sepsis. J Crit Care 49:92–98. doi:10.1016/j.jcrc.2018.10.028 30408726

[51] Segawa S, Kondo Y, Nakai Y, Iizuka A, Kaneko S, Yokosawa M, Furuyama K, Tsuboi H, Goto D, Matsumoto I, Sumida T. 2018. Placenta specific 8 suppresses IL-18 production through regulation of autophagy and is associated with adult still disease. J Immunol 201:3534–3545. doi:10.4049/jimmunol.1800667 30404814

[52] Schyns J, Bai Q, Ruscitti C, Radermecker C, De Schepper S, Chakarov S, Farnir F, Pirottin D, Ginhoux F, Boeckxstaens G, Bureau F, Marichal T. 2019. Non-classical tissue monocytes and two functionally distinct populations of interstitial macrophages populate the mouse lung. Nat Commun 10:3964. doi:10.1038/s41467-019-11843-0 31481690 PMC6722135

[53] Vazquez DE, Niño DF, De Maio A, Cauvi DM. 2015. Sustained expression of lipocalin-2 during polymicrobial sepsis. Innate Immun 21:477–489. doi:10.1177/1753425914548491 25227123

[54] Dwivedi A, Ui Mhaonaigh A, Carroll M, Khosravi B, Batten I, Ballantine RS, Hendricken Phelan S, O’Doherty L, George AM, Sui J, Hawerkamp HC, Fallon PG, Noppe E, Mason S, Conlon N, Ni Cheallaigh C, Finlay CM, Little MA, Bioresource OBOTSJATTARS. 2024. Emergence of dysfunctional neutrophils with a defect in arginase-1 release in severe COVID-19. JCI Insight 9:e171659. doi:10.1172/jci.insight.171659 39253969 PMC11385094

[55] Rios-Santos F, Alves-Filho JC, Souto FO, Spiller F, Freitas A, Lotufo CMC, Soares MBP, Dos Santos RR, Teixeira MM, Cunha F de Q. 2007. Down-regulation of CXCR2 on neutrophils in severe sepsis is mediated by inducible nitric oxide synthase-derived nitric oxide. Am J Respir Crit Care Med 175:490–497. doi:10.1164/rccm.200601-103OC 17138957

[56] Zhang Y, Zong L, Zheng Y, Zhang Y, Li N, Li Y, Jin Y, Chen L, Ouyang J, Bibi A, Huang Y, Xu Y. 2023. A single-cell atlas of the peripheral immune response in patients with influenza A virus infection. iScience 26:108507. doi:10.1016/j.isci.2023.108507 38089584 PMC10711475

[57] Liu X, Wang Y, Lu H, Li J, Yan X, Xiao M, Hao J, Alekseev A, Khong H, Chen T, et al.. 2019. Genome-wide analysis identifies NR4A1 as a key mediator of T cell dysfunction. Nature 567:525–529. doi:10.1038/s41586-019-0979-8 30814730 PMC6507425

[58] Wang L, Xiao Y, Luo Y, Master RP, Mo J, Kim M-C, Liu Y, Maharjan CK, Patel UM, De U, Carelock ME, Tithi TI, Li X, Shaffer DR, Guertin KR, Zhuang H, Moser E, Smalley KSM, Lv D, Zhou D, Zheng G, Zhang W. 2024. PROTAC-mediated NR4A1 degradation as a novel strategy for cancer immunotherapy. J Exp Med 221. doi:10.1084/jem.20231519 PMC10857906 38334978

[59] Zhu P, Wang J, Du W, Ren J, Zhang Y, Xie F, Xu G. 2022. NR4A1 promotes LPS-induced acute lung injury through inhibition of Opa1-mediated mitochondrial fusion and activation of PGAM5-related necroptosis. Oxid Med Cell Longev 2022:6638244. doi:10.1155/2022/6638244 35222801 PMC8881136

[60] Egarnes B, Blanchet MR, Gosselin J. 2017. Treatment with the NR4A1 agonist cytosporone B controls influenza virus infection and improves pulmonary function in infected mice. PLoS One 12:e0186639. doi:10.1371/journal.pone.0186639 29053748 PMC5650162

[61] Rüdiger D, Pelz L, Hein MD, Kupke SY, Reichl U. 2021. Multiscale model of defective interfering particle replication for influenza A virus infection in animal cell culture. PLoS Comput Biol 17:e1009357. doi:10.1371/journal.pcbi.1009357 34491996 PMC8448327

