# Supplementary material for: Single-cell and spatial transcriptomic profiling reveals distinct immune landscapes in murine lungs infected with H1N1 versus H5N1 influenza viruses
Source: J Virol. 2026 Jun 29;100(7):e00746-26. doi: 10.1128/jvi.00746-26 (PMC13386972; doi:10.1128/jvi.00746-26)
Supplement: Supplemental figures — Fig. S1 to S9. [file jvi.00746-26-s0001.pdf]

Supplementary information

Figure S1. Immune Cell Profiling in the Lungs of H1N1 and H5N1 Infected Mice.

(A) Violin plots showing the number of genes (nFeature_RNA), sequencing depth (nCount_RNA), and the percentage of mitochondrial genes (percent.mt) per sample in the control, H1N1-, and H5N1-infected groups at 1, 3, and 5 dpi before and after filtering the scRNA-seq data. (B) t-SNE visualization of the transcriptomic profiles of 84,162 cells. Each dot represents a single cell, colored according to its distinct transcriptional signature, which was clustered into 24 cell clusters. (C) t-SNE plots visualizing each cell subtype from individual samples of the control, H1N1-, and H5N1-infected groups, with distinct colors indicating different cell types. (D) Viral RNA reads from the single-cell RNA-seq dataset were mapped to the reference genomes of the H1N1 and H5N1 strains used in this study. Violin plots show the expression levels of the corresponding viral genomes detected in each annotated immune cell subset for mapping to H1N1 or H5N1 respectively. (E, F) Bar plots displaying the number of DEGs for each cell type in H1N1- and H5N1-infected mice compared to the control group, respectively. (G) GO biological process terms enriched by the differentially expressed genes across all cell types at 1, 3, and 5 dpi with H1N1 or H5N1 influenza virus. .

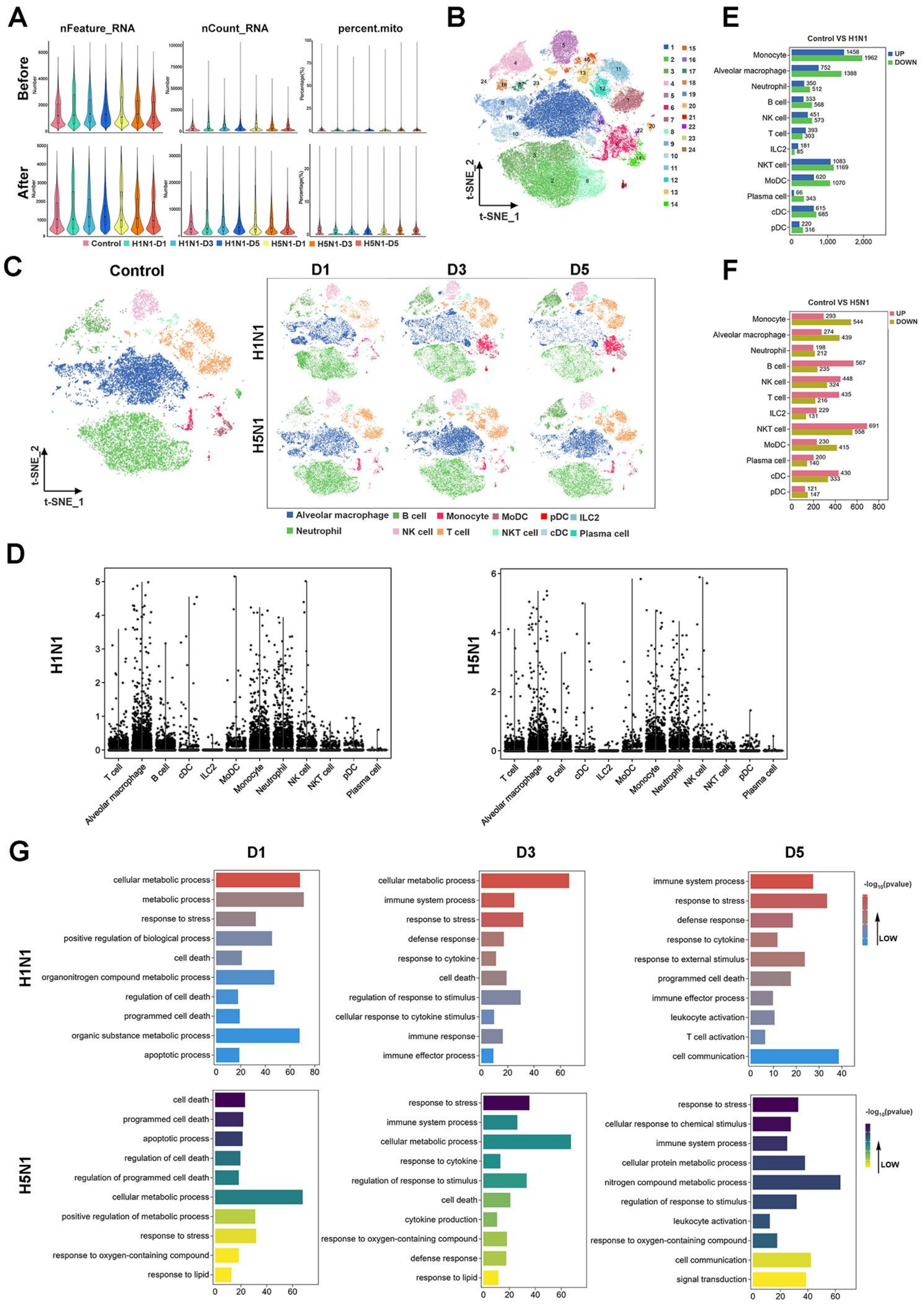

Figure S2. Flow Cytometric Analysis of Apoptosis and Necrosis in H1N1- or H5N1-Infected MH-S Cells.

(A) Representative flow cytometry plots from at least three independent experiments showing apoptosis and necrosis in MH-S cells 24 h post-infection with H1N1 or H5N1 at the indicated MOIs. (B) Quantification of apoptosis and necrosis rates. Data are shown as mean ± SD (n = 3). Significance is indicated as follows: *Control vs.

IAV; #H1N1 vs. H5N1.

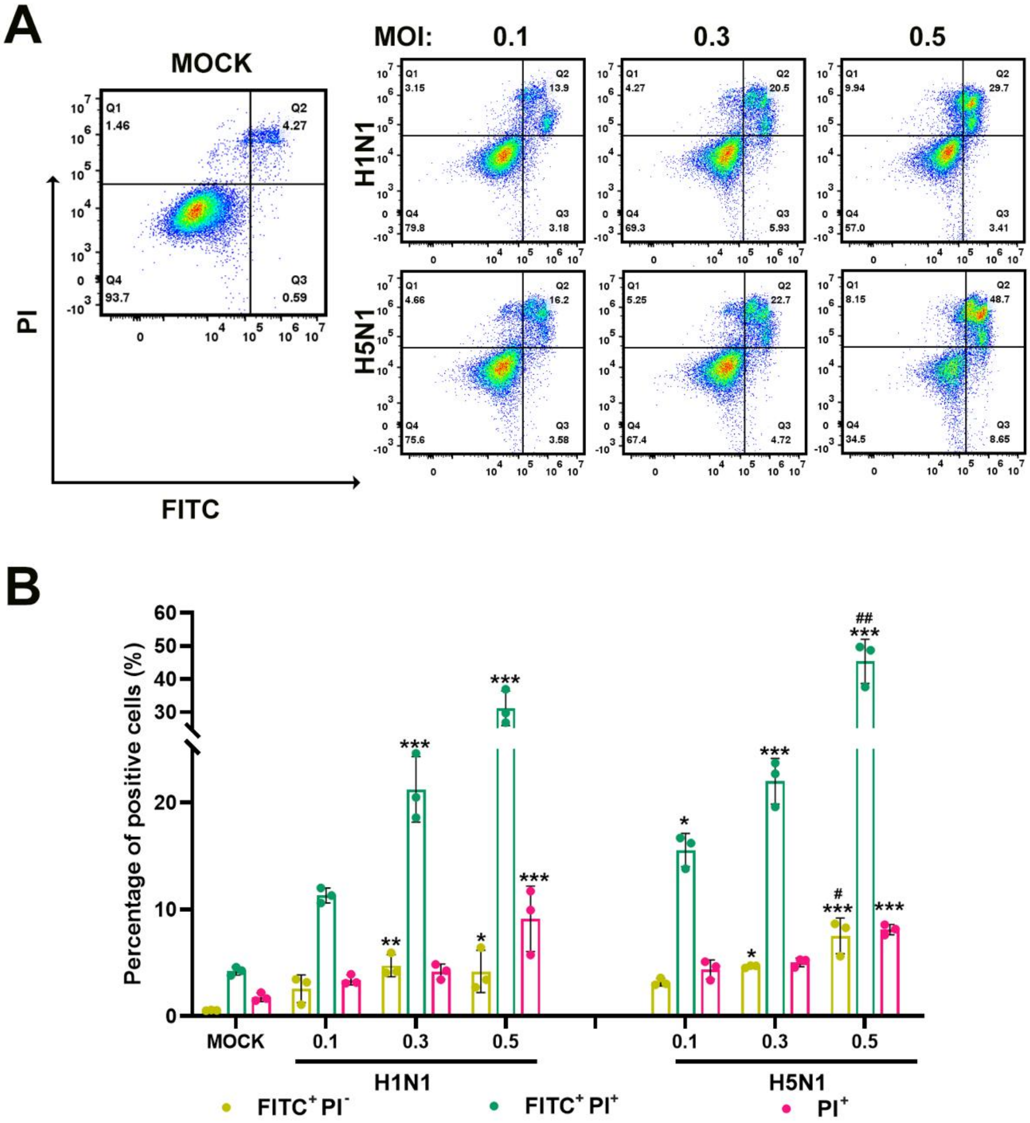

Figure S3. Cytokine and Cell Communication in Lungs of H1N1 and H5N1 Infected Mice.

(A, B) Heatmaps showing the expression levels of ISGs (A) and inflammatory cytokines (B) in alveolar macrophages, monocytes, and neutrophils from control, H1N1-, and H5N1-infected groups. (C) t-SNE plots depicting the expression levels of type I (Ifnar1, Ifnar2), type II (Ifngr1), and type III (Ifnlr1) interferon receptor subunits across all identified cell clusters. (D) Chord diagrams comparing the number of ligand-receptor pairs (left) and overall communication probability (right) across all

cell subpopulations in H1N1- and H5N1-infected mice relative to controls. Outer arcs are colored by cell subtype, with arc size representing the relative number of ligand-receptor pairs. Blue lines indicate stronger communication in controls, while red lines indicate stronger communication in the infected groups.

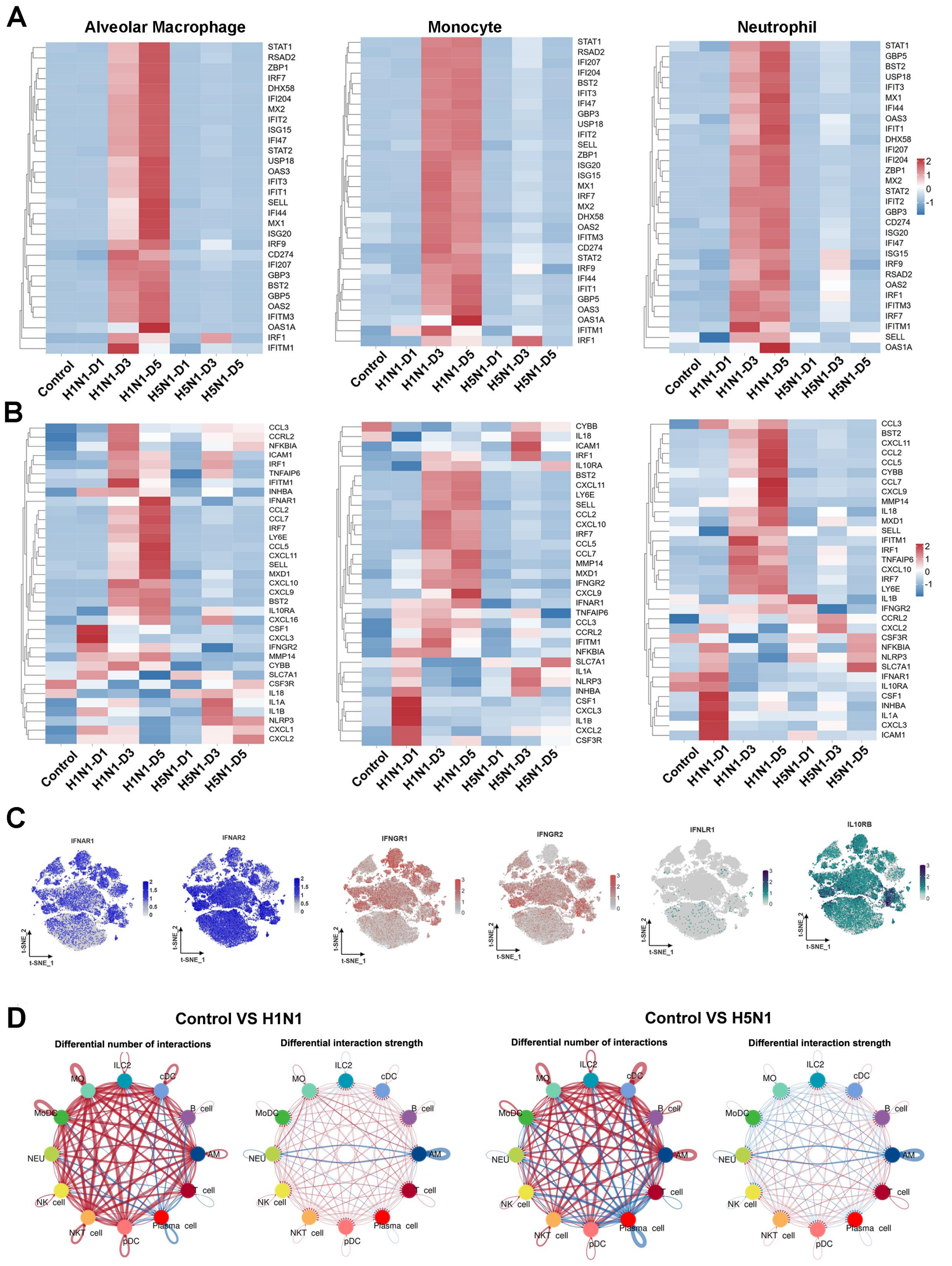

Figure S4. Analysis of Differentially Expressed Genes in Alveolar Macrophages in the IAV-infected Murine Lungs.

(A) Dot plot showing the canonical marker genes used to annotate the eight alveolar

macrophage (AM) subclusters. Dot size represents the percentage of cells expressing the gene, and color intensity represents the average expression level. (B) Scatter plots showing the expression dynamics of genes related to lipid metabolism (Lpl, Lpin1), inflammation (Il1b, S100a8), and interferon response (Isg15, Bst2) along the pseudotime trajectory. (C) Venn diagram (left) showing the overlap of upregulated DEGs in H1N1- and H5N1-infected mice compared to controls, and GO biological process terms (right) enriched in the uniquely upregulated genes from each infection. (D) KEGG pathway enrichment analysis of cell death-related DEGs across AM subclusters from H1N1- and H5N1-infected samples. The corresponding protein-protein interaction network was visualized with Cytoscape.

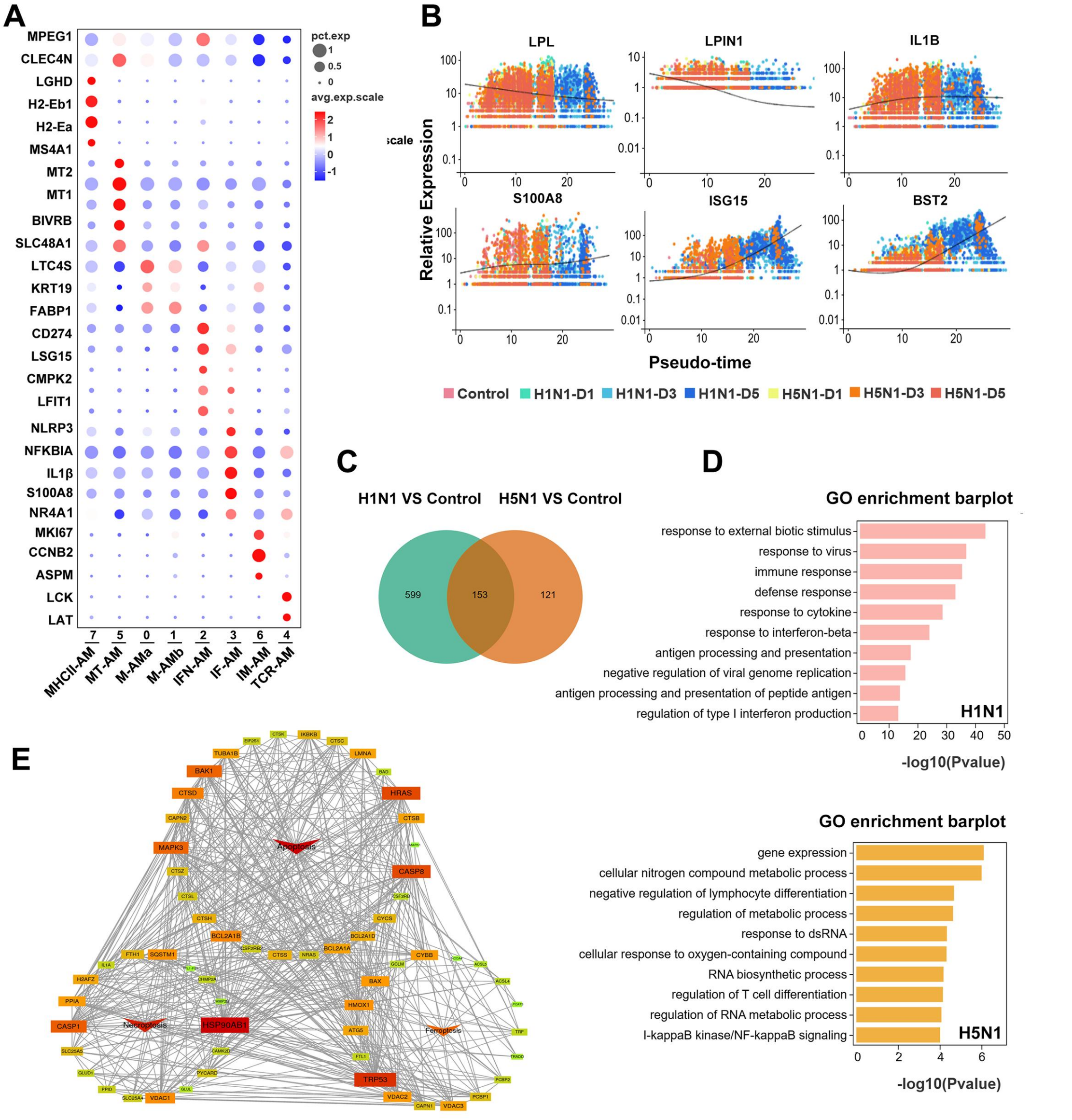

Figure S5. Identification of TCR-AM and AM-T Cell Communication in H1N1 vs. H5N1 Infection

(A) UMAP visualizations showing the expression of canonical AM marker genes (Chil3, Itgax; left) and T cell marker genes (Cd3d, Cd3e, Cd3g; right) across all AM subsets. The TCR-AM subset is circled. (B) Dot plot showing the expression of the indicated AM and T cell marker genes across AM subsets. (C) UMAP visualization showing the number of UMIs (nCount_RNA) across AM subsets, with the TCR-AM subset circled. (D) Violin plots comparing the number of genes detected

(nFeature_RNA), sequencing depth (nCount_RNA), and mitochondrial gene percentage (percent.mt) across all AM subsets. (E) Spatial communication between AMs and T cells mediated by the H2-D1-CD8B1 ligand-receptor pair was analyzed using CellTrek.

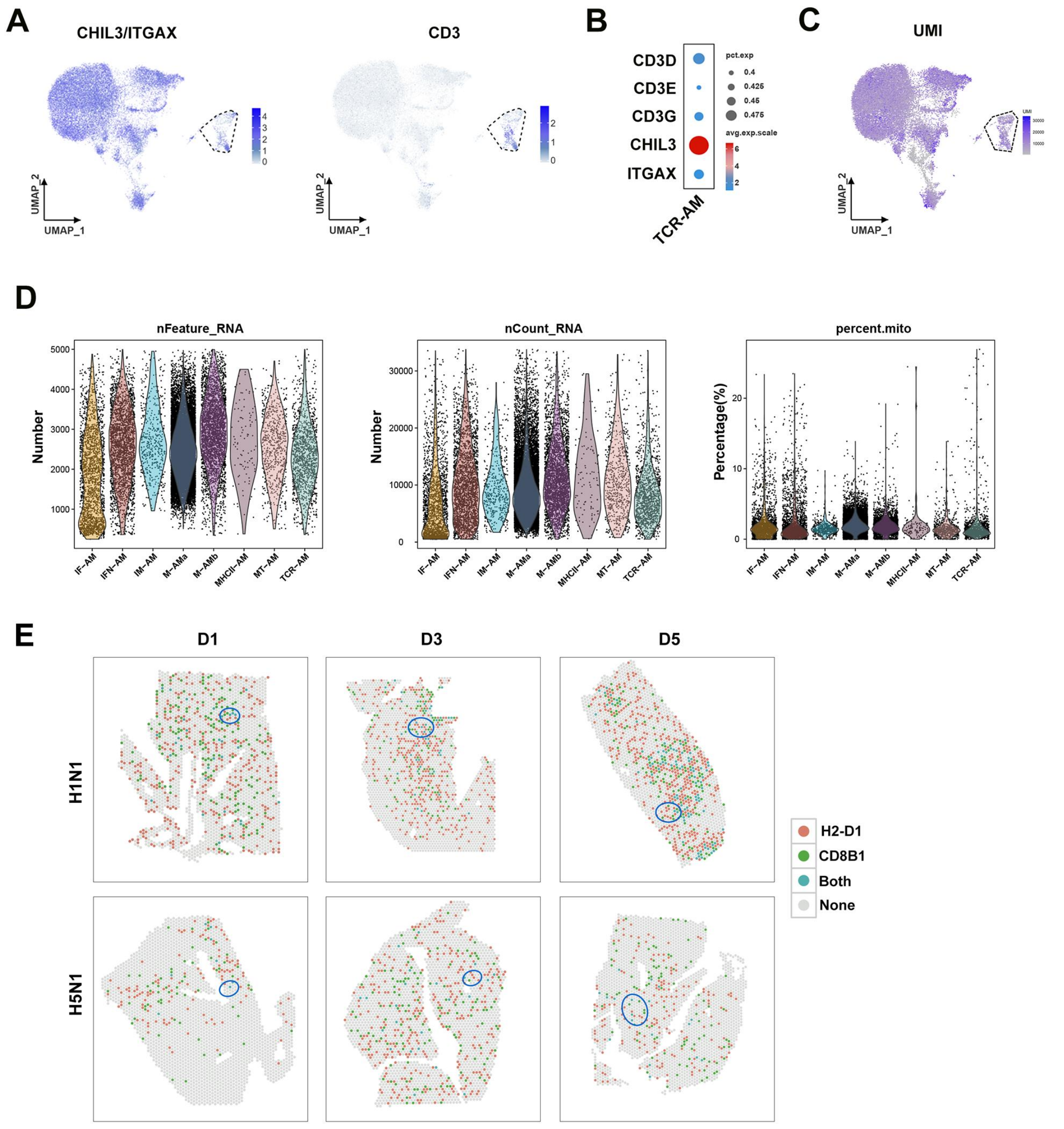

Figure S6. Analysis of Differentially Expressed Genes in Monocytes in the IAV-infected Murine Lungs.

(A) Dot plot showing the marker genes used for annotating the eight monocyte subclusters. (B) Dot plot showing the expression abundance of neutrophil activation-related genes in samples from the control, H1N1-, and H5N1-infected

groups. (C) Scatter plot illustrating the differentiation trajectory of classical monocytes (CMOs) and alveolar macrophages (upper right inset). Each dot represents a single cell, with numbers indicating branch points and colors corresponding to distinct differentiation states. Expression dynamics of inflammation- and lipid metabolism-associated markers (IL1B, S100A9, SPP1, LPL, LPIN1) across pseudotime. (D) Bar plot showing the number of DEGs in the CMO subset of H1N1- and H5N1-infected individuals compared to controls. (E) GO enrichment analysis of biological processes for the upregulated DEGs common to both H1N1 and H5N1 infections compared to the control group. (F) Heatmap showing the expression patterns of genes associated with the significantly enriched GO biological processes in samples from the control, H1N1-, and H5N1-infected groups.

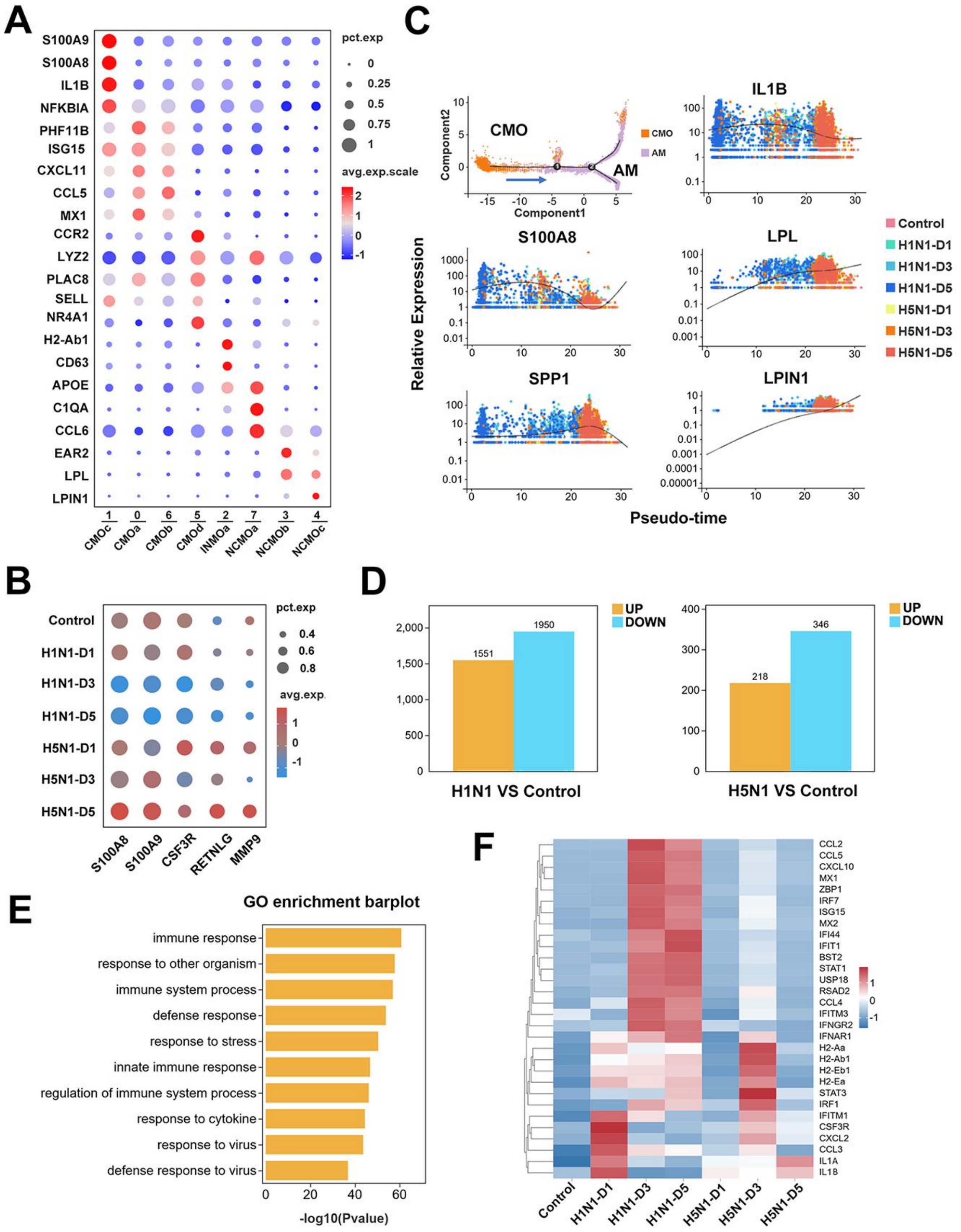

Figure S7. Analysis of Differentially Expressed Genes in Neutrophils in the IAV-infected Murine Lungs.

(A) Scatter plot depicting the expression dynamics of neutrophil development and differentiation markers (CXCR2, CSF3R, MMP8, MMP9, S100A8) along the pseudotime trajectory. (B) Number of up- and down-regulated DEGs in the IFN-NEU

subset of H1N1- and H5N1-infected groups versus the control group. (C) Significantly enriched GO biological processes for the upregulated DEGs in the IFN-NEU subset of H1N1- and H5N1-infected groups compared to the control.

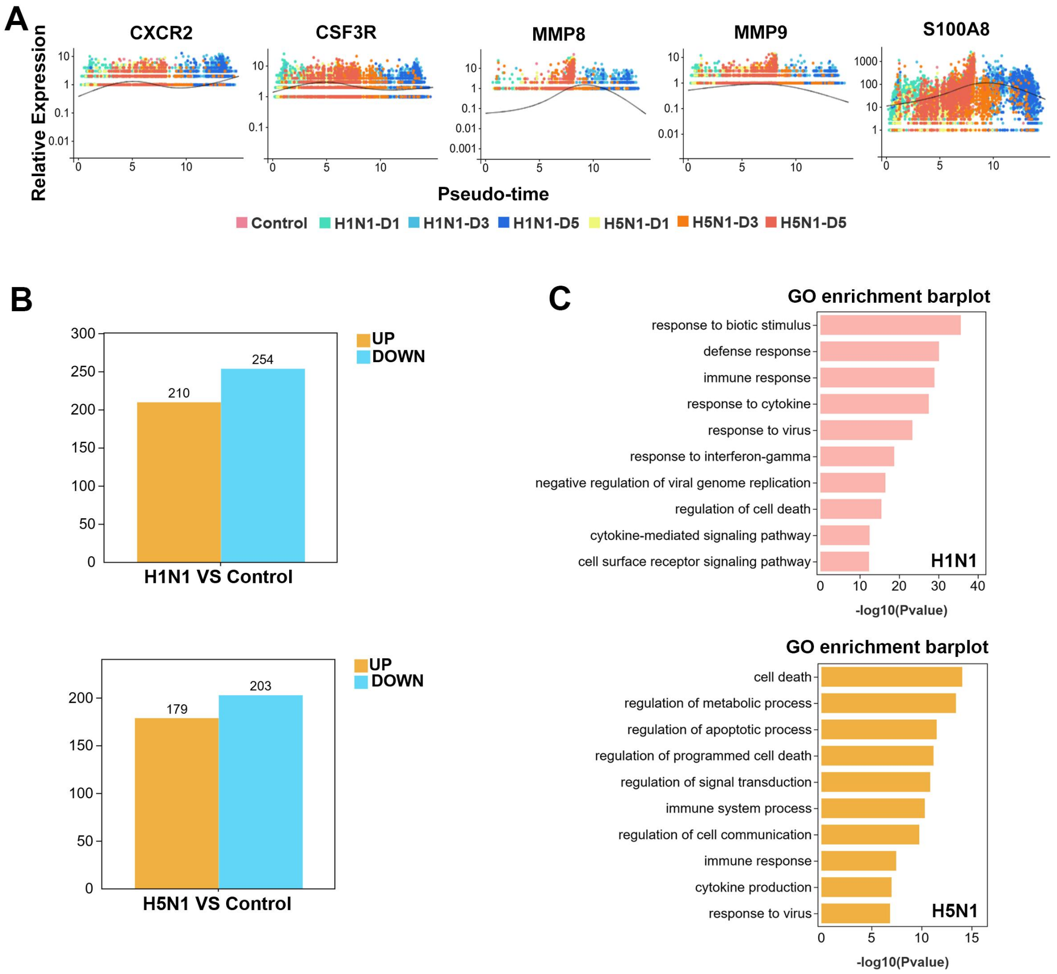

Figure S8. Flow Cytometry Analysis of Cellular Exhaustion Levels in the IAV-infected Murine Lungs. Gating strategy for three myeloid subsets: debris and doublets were excluded by FSC-A/SSC-A and FSC-H gates; $CD45^+$ immune cells were selected; alveolar macrophages ($CD11c^+$Siglec-F^+), monocytes ($CD11b^+Ly6C^+$), and neutrophils ($CD11b^+Ly6G^+$) were identified by sequential gating. (B) Quantification of alveolar macrophages, monocytes, and neutrophils in the lung tissues of control and virus-infected group mice. $^{**}p < 0.01$, Control vs. Treatment.

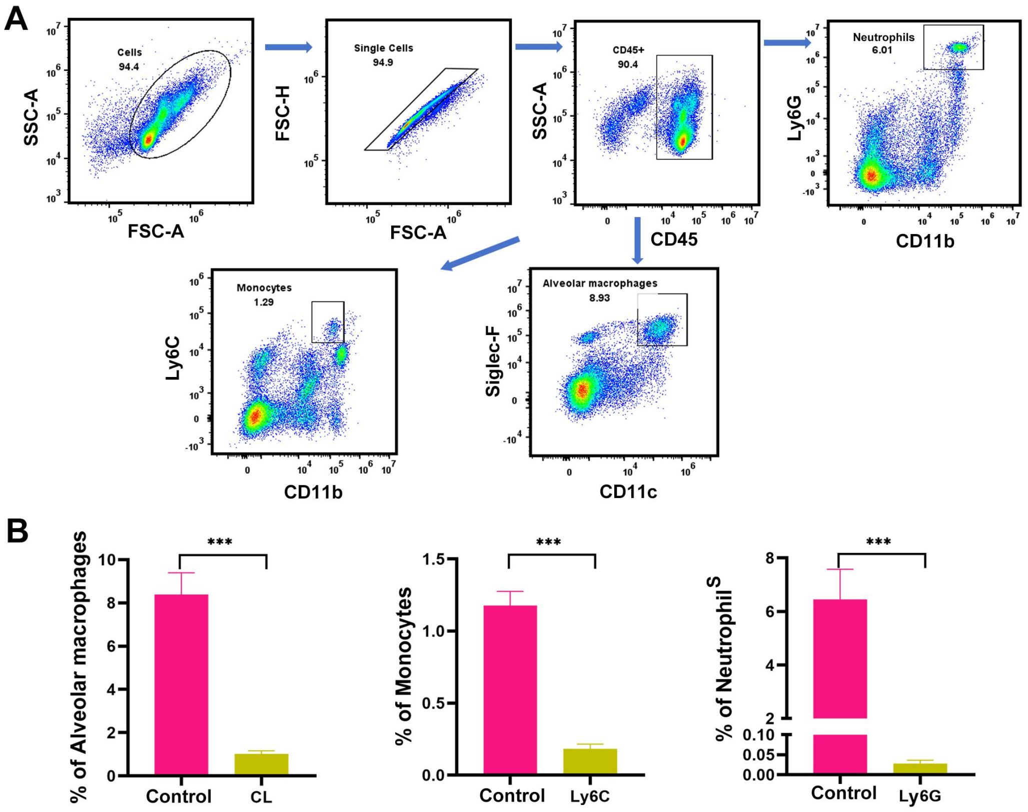

Figure S9. T Cell Subset Characterization and Intercellular Communication with Monocytes Following H1N1 and H5N1 Infection.

(A) Dot plot showing the expression levels of canonical T cell subset markers in the indicated cell populations from control, H1N1-, and H5N1-infected mice. (B) Stacked bar graph showing the relative frequencies of T cell subsets in control, H1N1-, and H5N1-infected mice. (C) Chord diagrams comparing the number of ligand-receptor pairs (left) and communication probability (right) across all cell subpopulations between H1N1- and H5N1-infected mice. Outer arcs are colored by cell subtype, with arc size representing the relative number of ligand-receptor pairs. Blue lines denote stronger communication in H1N1-infected mice, while red lines indicate stronger communication in H5N1-infected mice.

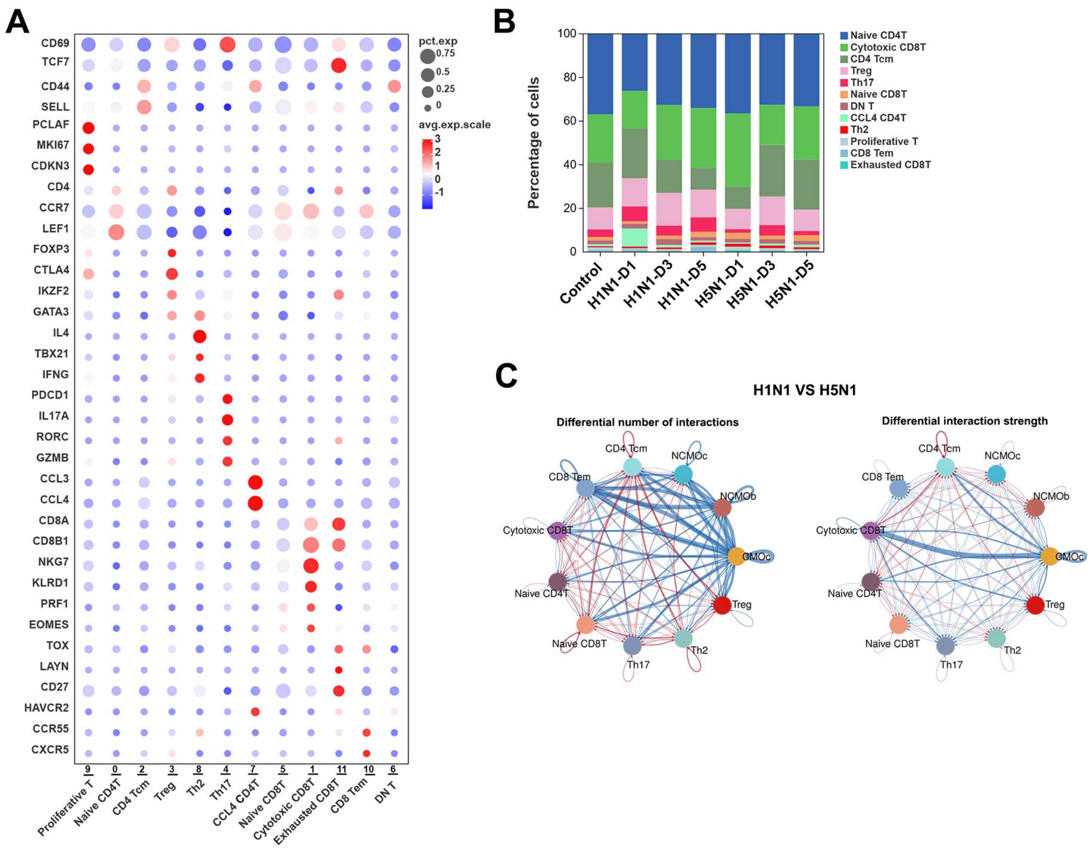
A
B
C
H1N1 VS H5N1
Differential number of interactions
Differential interaction strength
Percentage of cells
pct.exp
avg.exp.scale
Naive CD4T
Cytotoxic CD8T
CD4 Tcm
Treg
Th17
Naive CD8T
DN T
CCL4 CD4T
Th2
Proliferative T
CD8 Tem
Exhausted CD8T
Control
H1N1-D1
H1N1-D3
H1N1-D5
H5N1-D1
H5N1-D3
H5N1-D5
