# Supplementary material for: Single-cell and spatial transcriptomic profiling reveals distinct immune landscapes in murine lungs infected with H1N1 versus H5N1 influenza viruses
Source: J Virol. 2026 Jun 29;100(7):e00746-26. doi: 10.1128/jvi.00746-26 (PMC13386972; doi:10.1128/jvi.00746-26)
Supplement: Supplemental tables — Tables S1 to S7. [file jvi.00746-26-s0002.docx]

**Table S1. scRNA-seq data statistics**

| **Sample** | **Number of Reads** | **Valid Barcodes** | **Number of Cells (before_filter)** | **Number of Cells (after_filter)** | **Median UMI Counts per Cell** | **Median genes per Cell** | **Mapped to Genome** |
| --- | --- | --- | --- | --- | --- | --- | --- |
| **Control** | 644,822,900 | 97.7% | 14564 | 12778 | 3033 | 1203 | 93.5% |
| **H1N1-D 1** | 498,203,411 | 97.7% | 12659 | 11285 | 3393 | 1241 | 93.9% |
| **H1N1-D 3** | 454,501,842 | 98.2% | 13656 | 12126 | 3991 | 1347 | 92.2% |
| **H1N1-D 5** | 647,542,470 | 97.9% | 14184 | 12526 | 3624 | 1294 | 93.3% |
| **H5N1-D 1** | 428,532,023 | 98.1% | 11875 | 10669 | 2887 | 1092 | 92.4% |
| **H5N1-D 3** | 449,431,427 | 98.1% | 15447 | 13527 | 3756 | 1322 | 92.2% |
| **H5N1-D 5** | 376,959,803 | 98.1% | 12509 | 11251 | 2802 | 1090 | 92.3% |

**Table S2. Marker gene references for cell type annotation**

| **Cell subtype** | **Marker** | **References** |
| --- | --- | --- |
| **Neutrophil** | CXCR2, **S100A8**, **G0S2**， S100A12, **CSF3R** | PMID: 40091048 |
| **T/NK cell** | **CD3D**，**CD3E**，**CD3G**，**KLRD1**  **NKG7** |  |
| **Neutrophil** | **S100A8, S100A9, MMP9,** MMP8,**CSF3R,** IL1B | PMID: 32719519 |
| **DC** | CD74, LY6D, **BST2,** **RNASE6,**IRF8 |  |
| **Monocyte** | **MS4A4C,** APOE, **CCR2** |  |
| **B cell** | **CD79A**, **CD79B**, **EBF1**,CD74 |  |
| **T cell** | CD8B1, **CD3D, CD3G,** IL7R, LCK |  |
| **Monocyte** | **LY6C1**，**LY6C2**，ITGAM, CSF1R | PMID: 36260656 |
| **Alveolar macrophage** | **CD11C(ITGAX)**, **SIGLECF,** CD64, F4/80, MERTK, FABP4,MARCO, **CHIL3,** EAR1 | PMID: 36103853 |
| **pDC** | SIGLECH, LY6D, CCR9, COX6A2, PLAC8, **LY6C2**, TCF4, **BST2** |  |
| **cDC1/2** | **CCR7**, **CADM1**, **CD209A**, DPP4, ITGAE |  |
| **Alveolar macrophage** | PPAR-ɣ，**ABCG1** | PMID: 28185068 |
| **Plasma cell** | CD79A，**MZB1**，**SDC1**，**CD79B**，CD52 | PMID: 37138324 |
| **B cell** | **BLNK**, BTK, **CD19**, **CD79A**, **CD79B,** CR2, FCGR2B, IGHG3, IGHM | PMID: 34168645 |
| **NK cell** | **NCR1, NKG7,** KLRE1, **GZMA,** GZMB, **CCL5** | PMID: 35377804 |
| **B cell** | **CD79A**, MS4A1,**CD79B**, IGHD, **CD19** | PMID: 29775597 |
| **NK Cell** | **NKG7**, KLRA8, KLRA4, KLRB1C |  |
| **pDC** | MS4A6C, PLAC8, **BST2,** IRF7, IRF5 |  |
| **T cell** | **TRBC2,** CD8B1, **CD3D, CD3G,**THY1 |  |
| **Alveolar macrophage** | EAR2, EAR1, CD68, MARCO, **SIGLECF** |  |
| **DC** | CD74, **CD83**, **CD209A**,**CCR7**, CST3 |  |
| **DC** | **TMEM123**, CD40, CD80 | PMID: 20685650 |
| **DC** | **CCL22**, **CD209A**, ITGAE | PMID: 38348034 |
| **ILC2** | **IL1RL1**, GATA3 | PMID: 32673538 |
| **ILC** | **CSF2**, GATA3, GFI1, IRGAE, RORC | PMID: 32373555 |
| **monocyte** | **ENO3**, **CD300E**, AGPAT4, RBPMS, SLC12A2 | PMID: 41252214 |
| **MoDC** | **CD143(ACE)**,CD80, CD40, CD86 | PMID: 14662338 |

**Table S3. ST data statistics**

| **Sample** | **Number of Reads** | **Valid Barcodes** | **Sequencing Saturation** | **Number of Spots Under Tissue** | **Median UMI Counts per Spot** | **Median Genes per Spot** |
| --- | --- | --- | --- | --- | --- | --- |
| **Control** | 304,292,781 | 98.64% | 89.35% | 2012 | 11906 | 4742 |
| **H1N1-D 1** | 322,961,486 | 98.68% | 77.75% | 2412 | 21635.5 | 6360.5 |
| **H1N1-D 3** | 183,789,855 | 97.80% | 89.33% | 2849 | 3997 | 2169 |
| **H1N1-D 5** | 403,992,619 | 98.49% | 93.21% | 1896 | 12426.5 | 4718.5 |
| **H5N1-D 1** | 304,667,143 | 98.57% | 88.11% | 2595 | 4599 | 2435 |
| **H5N1-D 3** | 374,017,949 | 98.70% | 86.46% | 2533 | 15669 | 5571 |
| **H5N1-D 5** | 306,309,820 | 98.71% | 81.08% | 2572 | 18338 | 6152.5 |

**Table S4.** **Number of cells in each cluster and their proportional distribution in each group**

| **Cluster** | **Control** | **H1N1-D1** | **H1N1-D 3** | **H1N1-D 5** | **H5N1-D 1** | **H5N1-D 3** | **H5N1-D 5** |
| --- | --- | --- | --- | --- | --- | --- | --- |
| **Total** | 12778(100%) | 11285(100%) | 12126(100%) | 12526(100%) | 10669(100%) | 13527(100%) | 11251(100%) |
| **1** | 4175 (32.67%) | 2129 (18.87%) | 1660(13.69%) | 758 (6.05%) | 2169 (20.33%) | 4079 (30.15%) | 2842(25.26%) |
| **2** | 1985 (15.53%) | 2019 (17.89%) | 1564 (12.9%) | 1127 (9%) | 2210 (20.71%) | 2053 (15.18%) | 1749(15.55%) |
| **3** | 1287 (10.07%) | 2029 (17.98%) | 1145 (9.44%) | 809 (6.46%) | 1268 (11.88%) | 1351 (9.99%) | 1055 (9.38%) |
| **4** | 580 (4.54%) | 487 (4.32%) | 897 (7.4%) | 1245 (9.94%) | 504 (4.72%) | 512 (3.79%) | 1065 (9.47%) |
| **5** | 534 (4.18%) | 545 (4.83%) | 616 (5.08%) | 1728 (13.8%) | 430 (4.03%) | 672 (4.97%) | 328 (2.92%) |
| **6** | 345 (2.7%) | 239 (2.12%) | 1146 (9.45%) | 1711(13.66%) | 312 (2.92%) | 280 (2.07%) | 221 (1.96%) |
| **7** | 541 (4.23%) | 219 (1.94%) | 539 (4.44%) | 790 (6.31%) | 604 (5.66%) | 692 (5.12%) | 819 (7.28%) |
| **8** | 657 (5.14%) | 604 (5.35%) | 676 (5.57%) | 407 (3.25%) | 625 (5.86%) | 660 (4.88%) | 534 (4.75%) |
| **9** | 679 (5.31%) | 564 (5%) | 733 (6.04%) | 535(4.27%) | 665 (6.23%) | 603 (4.46%) | 257 (2.28%) |
| **10** | 148 (1.16%) | 1019 (9.03%) | 931 (7.68%) | 573 (4.57%) | 431 (4.04%) | 258 (1.91%) | 130 (1.16%) |
| **11** | 329 (2.57%) | 143 (1.27%) | 431 (3.55%) | 626 (5%) | 536 (5.02%) | 394 (2.91%) | 579 (5.15%) |
| **12** | 285 (2.23%) | 227 (2.01%) | 253 (2.09%) | 207 (1.65%) | 140 (1.31%) | 476 (3.52%) | 492 (4.37%) |
| **13** | 216 (1.69%) | 182 (1.61%) | 370 (3.05%) | 454 (3.62%) | 166 (1.56%) | 384 (2.84%) | 287 (2.55%) |
| **14** | 257 (2.01%) | 87 (0.77%) | 288 (2.38%) | 420 (3.35%) | 99 (0.93%) | 108 (0.8%) | 106 (0.94%) |
| **15** | 79 (0.62%) | 136 (1.21%) | 203 (1.67%) | 338 (2.7%) | 114 (1.07%) | 193 (1.43%) | 164 (1.46%) |
| **16** | 191 (1.49%) | 250 (2.22%) | 59 (0.49%) | 72 (0.57%) | 118 (1.11%) | 209 (1.55%) | 132 (1.17%) |
| **17** | 95 (0.74%) | 92 (0.82%) | 142 (1.17%) | 210 (1.68%) | 39 (0.37%) | 147 (1.09%) | 163 (1.45%) |
| **18** | 67 (0.52%) | 137 (1.21%) | 138 (1.14%) | 119 (0.95%) | 15 (0.14%) | 143 (1.06%) | 155 (1.38%) |
| **19** | 127 (0.99%) | 31 (0.27%) | 109 (0.9%) | 43 (0.34%) | 105 (0.98%) | 100 (0.74%) | 17 (0.15%) |
| **20** | 89 (0.7%) | 35 (0.31%) | 34 (0.28%) | 14 (0.11%) | 66 (0.62%) | 109 (0.81%) | 59 (0.52%) |
| **21** | 12 (0.09%) | 10 (0.09%) | 91 (0.75%) | 192 (1.53%) | 9 (0.08%) | 17 (0.13%) | 23 (0.2%) |
| **22** | 28 (0.22%) | 35 (0.31%) | 65 (0.54%) | 97 (0.77%) | 22 (0.21%) | 25 (0.18%) | 37 (0.33%) |
| **23** | 64 (0.5%) | 54 (0.48%) | 31 (0.26%) | 37 (0.3%) | 20 (0.19%) | 57 (0.42%) | 32 (0.28%) |
| **24** | 8 (0.06%) | 12 (0.11%) | 5 (0.04%) | 14 (0.11%) | 2 (0.02%) | 5 (0.04%) | 5 (0.04%) |

| **Cluster** | **Signature genes** | **Cell type annotation** |
| --- | --- | --- |
| **Cluster 1/9/10/16/19** | CHIL3,Siglec-F,ITGAX,ABCG1 | Alveolar macrophage |
| **Cluster 2/3/8/20** | MMP9, CSF3R,S100A8,S100A9,G0S2 | Neutrophil |
| **Cluster 4/17/18** | CD19,CD79A,CD79B,EBF1 | B cell |
| **Cluster 24** | CD79B,BLNK,MZB1,SDC1 | Plasma cell |
| **Cluster 5** | NKG7,NCR1,KLRD1,GZMA,CCL5 | NK cell |
| **Cluster 15** | GZMA,NCR1,CCL5,CD3G,TRBC2 | NKT cell |
| **Cluster 7/11/12/13** | CD3D,CD3E,CD3G,TRBC2 | T cell |
| **Cluster 6** | Ly6C1,Ly6C2,CCR2,MS4A6C | Monocyte |
| **Cluster 21** | Ly6C2,BST2,CADM1,RNASE6 | pDC |
| **Cluster 14** | ACE,CD300E,ADGRE4,ENO3 | MoDC |
| **Cluster 22** | CD209A,CCR7,CD83,TMEM123,CCL22 | cDC |
| **Cluster 23** | STAB2,CCDC184,CSF2,IL1RL1 | ILC2 |

**Table S5. Cell type annotation based on the marker genes information**

**Table S6 . Inflammatory gene and IFN score, related to Figure 3**

| **Inflammatory genes** | **ISG** |
| --- | --- |
| ABCA1, ABI1, ACVR1B, ACVR2A, ADGRE1, ADM, ADORA2B, ADRM1, AHR, ALDOA, APLNR, AQP9, ARHGAP26, ATP2A2, ATP2B1, ATP2C1, ATP5O, AXL, BDKRB1, BEST1, BST2, BTG2, C3AR1, C5AR1, CALCRL, CCL17, CCL2, CCL20, CCL22, CCL24, CCL3, CCL4，CCL5, CCL7, CCR7, CCRL2, CD14, CD40, CD48, CD55, CD69, CD70, CD82, CDKN1A, CHST2, CLEC5A, CMKLR1, CRP, CSF1, CSF3, CSF3R, CX3CL1, CXCL1, CXCL10, CXCL11  CXCL16, CXCL2, CXCL3, CXCL9, CXCR6, CYBB, CBLD2  DDIT3, DPEP2, EBI3, EDN1, EIF2AK2, EMP3, EREG, F3  FAM220A, FFAR2, FPR1, FZD5, GABBR1, GCAT, GCH1  GNA15, GNAI3, GP1BA, GPC3, GPR132, GPR183, HAS2  HBEGF, HIF1A, HPN, HRH1, ICAM1, ICAM4, IFITM1, IFNAR1, IFNGR2, IL10, IL10RA, IL12B, IL15, IL15RA, IL18, IL18R1, IL18RAP, IL1A, IL1B, IL1R1, IL2RB, IL6, IL7R, INHBA, IRAK2, IRF1, IRF7, ITGA5, ITGB3, ITGB8, KCNA3, KCNJ2, KCNMB2, KIF1B, KLF6, LAMP3, LCK, LCP2, LDLR, LHB, LIF, LPAR1, LTA, LY6E, LYN, MARCO, MEFV, MEP1A, MET, MMP14, MSR1, MXD1, MYC, NAMPT, NDOR1, NDP, NFKB1, NFKBIA, NLRP3, NMI, NMUR1, NNT, NOD2, NPFFR2, NRG1, OLFR290, OLR1, OPRK1, OSM, OSMR, P2RX4, P2RX7, P2RY2, PAKAP, PCDH7, PCDHA11, PDE4B, PDPN, PIK3R5, PLAUR, PROK2, PSEN1, PTAFR, PTGER2, PTGER4, PTGIR, PTP4A1, PTPRE, PVR, RAF1, RASGRP1, RELA, RGS1, RGS16, RHOG, RIPK2, RNF144B,ROS1, RTP4, SCARF1, SCN1B, SELE, SELENOS, SELL, SEMA4D, SEPTIN2, SERPINE1, SGMS2, SLAMF1, SLC11A2, SLC1A2, SLC28A2, SLC31A1, SLC31A2, SLC4A4, SLC7A1, SLC7A2, SRI, STAB1, TACR1, TACR3, TAPBP, TIMP1, TLR1, TLR2, TLR3, TNFAIP6, TNFRSF1B, TNFRSF9, TNFSF10, TNFSF14, TNFSF15, TNFSF9, VIP | ADAR, AIDA, ANKFY1, BST2, CASP4, CCRL2, CD274, CD69  CD86, CMPK2, CNP, CXCL10, DCK, DDX58, DDX60, DHX58  DTX3L, EHD4, EPSTI1, GBP2, GBP3, GBP4, GBP5, GCH1  IFI202B, IFI203, IFI204, IFI205, IFI206, IFI207, IFI208, IFI209  IFI211, IFI214, IFI27L2A, IFI35, IFI44, IFI44L, IFI47, IFIH1,  IFIT1, IFIT1BL1, IFIT2, IFIT3, IFIT3B, IFITM1, IFITM3, IL15RA, IL1RN, IRF1, IRF7, IRF9, ISG15, ISG20, LGALS3BP  LGALS9, MAX, MOV10, MX1, MX2, NAMPT, NLRC5, NMI  OAS1A, OAS2, OAS3, OASL1, OASL2, OGFR, PARP11, PARP12, PARP14, PARP9, PCGF5, PML, PNPT1, PPA1, PSME2, RBM43, RNF213, RSAD2, RTP4, SAMD9L, SELL, SLFN5, SP110, STAT1, STAT2, TAP1, TDRD7, TLK2, TMEM140, TRAFD1, TRIM21, UBE2L6, USP18, USP25, VCPIP1, XAF1, ZBP1, ZCCHC2, ZNFX1 |

**Table S7.** **Alveolar macrophage counts and frequencies per cluster and group**

| **Cluster** | **Control** | **H1N1-D1** | **H1N1-D 3** | **H1N1-D 5** | **H5N1-D 1** | **H5N1-D 3** | **H5N1-D 5** |
| --- | --- | --- | --- | --- | --- | --- | --- |
| **Total** | 5320(100%) | 3993(100%) | 3492(100%) | 1981(100%) | 3488(100%) | 5249(100%) | 3378(100%) |
| **0** | 3682 (69.21%) | 2744 (68.72%) | 1358(38.89%) | 602 (30.39%) | 2396 (68.69%) | 3614 (68.85%) | 2662 (78.8%) |
| **1** | 604 (11.35%) | 518(12.97%) | 394 (11.28%) | 225 (11.36%) | 550 (15.77%) | 561(10.69%) | 262(7.76%) |
| **2** | 192(3.61%) | 142 (3.56%) | 1015(29.07%) | 608 (30.69%) | 129 (3.7%) | 155 (2.95%) | 110 (3.26%) |
| **3** | 422 (7.93%) | 291 (7.29%) | 272 (7.79%) | 173 (8.73%) | 160 (4.59%) | 413 (7.87%) | 179 (5.3%) |
| **4** | 182 (3.42%) | 73 (1.83%) | 281 (8.05%) | 282 (14.24%) | 92 (2.64%) | 283 (5.39%) | 62 (1.84%) |
| **5** | 101 (1.9%) | 170 (4.26%) | 62 (1.78%) | 44 (2.22%) | 70 (2.01%) | 123 (2.34%) | 75 (2.22%) |
| **6** | 114 (2.14%) | 35 (0.88%) | 94 (2.69%) | 40 (2.02%) | 84 (2.41%) | 81 (1.54%) | 18 (0.53%) |
| **7** | 23 (0.43%) | 20 (0.5%) | 16 (0.46%) | 7 (0.35%) | 7 (0.2%) | 19 (0.36%) | 10 (0.3%) |
